# Longitudinal Associations of Magnetic Susceptibility with Clinical Severity in Parkinson's Disease

**DOI:** 10.1002/mds.29702

**Published:** 2024-01-03

**Authors:** George E.C. Thomas, Naomi Hannaway, Angelika Zarkali, Karin Shmueli, Rimona S. Weil

**Affiliations:** ^1^ Dementia Research Centre UCL Institute of Neurology London UK; ^2^ Department of Medical Physics and Biomedical Engineering University College London London UK; ^3^ Wellcome Centre for Human Neuroimaging University College London London UK; ^4^ Movement Disorders Consortium University College London London UK

**Keywords:** Parkinson's disease, dementia, MRI, susceptibility, QSM, longitudinal

## Abstract

**Background:**

Dementia is common in Parkinson's disease (PD), but there is wide variation in its timing. A critical gap in PD research is the lack of quantifiable markers of progression, and methods to identify early stages of dementia. Atrophy‐based magnetic resonance imaging (MRI) has limited sensitivity in detecting or tracking changes relating to PD dementia, but quantitative susceptibility mapping (QSM), sensitive to brain tissue iron, shows potential for these purposes.

**Objective:**

The objective of the paper is to study, for the first time, the longitudinal relationship between cognition and QSM in PD in detail.

**Methods:**

We present a longitudinal study of clinical severity in PD using QSM, including 59 PD patients (without dementia at study onset), and 22 controls over 3 years.

**Results:**

In PD, increased baseline susceptibility in the right temporal cortex, nucleus basalis of Meynert, and putamen was associated with greater cognitive severity after 3 years; and increased baseline susceptibility in basal ganglia, substantia nigra, red nucleus, insular cortex, and dentate nucleus was associated with greater motor severity after 3 years. Increased follow‐up susceptibility in these regions was associated with increased follow‐up cognitive and motor severity, with further involvement of hippocampus relating to cognitive severity. However, there were no consistent increases in susceptibility over 3 years.

**Conclusions:**

Our study suggests that QSM may predict changes in cognitive severity many months prior to overt cognitive involvement in PD. However, we did not find robust longitudinal changes in QSM over the course of the study. Additional tissue metrics may be required together with QSM for it to monitor progression in clinical practice and therapeutic trials. © 2024 The Authors. *Movement Disorders* published by Wiley Periodicals LLC on behalf of International Parkinson and Movement Disorder Society.

Although considered a movement disorder, Parkinson's disease (PD) causes significant non‐motor symptoms, of which dementia and cognitive changes are among the most common and distressing.^1^ With the emergence of potentially disease‐modifying therapies for neurodegeneration,^2^, ^3^ there is increasing pressure to start treatment at earlier stages. Although no such medication exists yet for PD, being able to detect changes relating to neurodegeneration at earlier stages of PD dementia may enable patients to be identified for future treatments, and allow measuring and monitoring of outcomes. Until recently, neuroimaging using magnetic resonance imaging (MRI) has shown only limited potential in detecting the early stages of PD progression, mainly because atrophy‐based approaches are relatively insensitive in this context.^4^ Instead, measures sensitive to changes in tissue composition are more likely to detect and track neurodegeneration in PD. Magnetic susceptibility measured using quantitative susceptibility mapping (QSM) is increased in relevant brain regions in patients with poorer cognition and worse motor scores in cross‐sectional studies.^5^, ^6^, ^7^ Measuring brain tissue iron is of particular relevance in pathological progression in PD, as iron accumulates in the basal ganglia and substantia nigra (SN),^8^, ^9^ and excess tissue iron causes an increase in reactive oxygen species^10^ that interact with alpha‐synuclein^11^ and beta‐amyloid.^12^ We recently showed that brain vulnerability to neurodegeneration in PD relates to regional differences in gene expression for genes relating to heavy metal metabolism, including iron.^13^

However, it is not yet established whether QSM can detect brain tissue changes in patients who worsen cognitively over time, or whether it can track longitudinal changes relating to neurodegeneration.

So far, there have been few longitudinal QSM studies in PD, with none specifically examining progression of cognitive decline. Three studies have investigated changes in susceptibility in pre‐selected regions of interest (ROIs) over time, with a primary focus on the SN and relationships with motor severity.^14^, ^15^, ^16^ Du et al reported decreasing susceptibility in the SN pars reticulata, but not the pars compacta, over 18 months in late‐stage PD.^14^ Guan et al also reported decreased susceptibility in PD patients with motor asymmetry in the SN, globus pallidus, and red nucleus over 18 months.^16^ Conversely, Bergsland et al reported increased susceptibility in the ventral SN over 3 years in people with PD relative to controls.^15^

Here, we identify susceptibility changes relating to cognitive severity in people with PD followed longitudinally for 3 years, using a voxel‐wise whole‐brain approach as well as ROI analysis. We hypothesized that increased susceptibilities in relevant brain regions at baseline would be associated with poorer future cognitive and motor performance in people with PD. We also hypothesized that there would be overall increases in QSM values during the disease course in PD (beyond what would be expected with normal aging), particularly in iron‐rich deep‐brain nuclei.

## Patients and Methods

### Participants

A total of 107 patients with PD within 10 years of diagnosis were recruited to this study between October 2017 and December 2018, as described previously.^5^ Inclusion criteria were clinically diagnosed, early to mid‐stage PD (Queen Square Brain Bank Criteria) aged 49–80 years. Exclusion criteria were confounding neurological or psychiatric disorders, dementia, and contraindications to MRI. Participants continued their usual therapy (including levodopa [l‐dopa]) for all assessments and scans; none were taking cholinesterase inhibitors. A total of 37 age‐matched controls were recruited from sources, including unaffected patient spouses. Participants were invited back 3 years later with a follow‐up interval of 38.5 ± 4.4 months (mean ± SD). Of the 144 (107 PD) participants seen at baseline, 89 (67 PD) were available and eligible at follow‐up. After quality control of MRI images (by a rater blind to clinical outcomes), 8 PD participants were excluded. Thus, 81 (59 PD) participants were included in the current analysis. A flowchart detailing reasons for participant exclusion can be seen in Figure S1. All participants provided written informed consent, and the study was approved by the Queen Square Research Ethics Committee.

### Clinical Assessments

All participants underwent detailed clinical assessments at both time points, as described previously.^17^ Cognitive scores across five domains (Stroop color, Hooper visual organization, word recognition, verbal fluency category, verbal fluency letter) as well as the Montreal Cognitive Assessment (MoCA) score were combined into a combined cognitive score using z‐scores based on control means and standard deviations.^18^ As this combined score is based on a more detailed assessment, it is likely to have greater variance and sensitivity to differences between individuals than the MoCA alone. Motor function was assessed using the Movement Disorder Society Unified Parkinson's Disease Rating Scale Part III (MDS‐UPDRS III) with patients in the “On” state. Additional tests including smell, depression and anxiety, and rapid eye movement (REM) sleep behavioral disorder questionnaires were also carried out. Participant demographics and clinical measures are presented in Table 1.

**TABLE 1:** Demographics table for participants included in longitudinal analysis

Measure	Controls (N = 22)	Parkinson's disease (N = 59)	Stat	*P*
Sex (F/M)	12/10	28/31	OR = 1.33	0.62
Age (y)				
Baseline	66.7 (8.7)	63.0 (7.2)	*T* = 1.93	0.057
Follow‐up	69.9 (8.8)	66.2 (7.1)	*T* = 1.95	0.055
Years of education	18.0 (1.8)	17.2 (2.7)	*U* = 994	0.32
MoCA score				
Baseline	29.1 (1.1)	28.4 (1.7)	*U* = 1054	0.094
Follow‐up	29.0 (1.5)	27.8 (2.8)	*U* = 1061	0.082
Combined cognitive score				
Baseline	0.11 (0.57)	−0.11 (0.74)	*U* = 1004	0.28
Follow‐up	0.07 (0.49)	−0.23 (0.91)	*U* = 1003	0.29
UPDRS‐III				
Baseline	6.1 (5.0)	22.3 (12.3)	*T* = −6.01	5.4 × 10^−8^
Follow‐up	5.2 (4.2)	28.7 (9.1)	*U* = 263.5	1.2 × 10^−11^
HADS depression				
Baseline	1.0 (1.2)	3.4 (2.7)	*U* = 524.5	4.7 × 10^−5^
Follow‐up	2.0 (2.6)	4.3 (2.7)	*U* = 565.5	3.2 × 10^−4^
HADS anxiety				
Baseline	3.5 (3.7)	5.8 (3.9)	*U* = 666.5	0.012
Follow‐up	3.6 (3.8)	5.8 (3.9)	*U* = 640	0.0052
RBDSQ score				
Baseline	2.3 (1.4)	3.8 (2.3)	*U* = 669	0.012
Follow‐up	1.6 (1.5)	4.7 (3.0)	*U* = 468.5	3.6 × 10^−6^
Disease duration (y)				
Baseline	‐	4.1 (2.6)	‐	‐
Follow‐up	‐	7.3 (2.7)	‐	‐
LEDD (mg)				
Baseline	‐	432.1 (228.0)	‐	‐
Follow‐up	‐	651.2 (354.7)	‐	‐
Motor deficit dominance (left/right/both)*				
Baseline	‐	23/32/4	OR = 0.72	0.27
Follow‐up	‐	24/27/8	OR = 0.89	0.78

*Note*: Means (SDs) reported.

Abbreviations: MoCA, Montreal Cognitive Assessment; HADS, Hospital Anxiety and Depression Scale; RBDSQ, REM (Rapid Eye Movement) Sleep Behavior Disorder Screening Questionnaire; LEDD, levodopa equivalent dose; Statistical tests: OR, Fisher's exact test odds ratio; *T*, two‐tailed *t* test statistic; *U*, Mann–Whitney *U*‐test test statistic; UPDRS‐III, Unified Parkinson's Disease Rating Scale Part 3 (motor score).

* For motor deficit dominance, the actual left/right ratio is compared against the null hypothesis of equal left/right ratio.

### MRI Protocol

At both time points, MRI measurements were performed on the same Siemens Prisma‐fit 3 T MRI system using a 64‐channel receive array coil (Siemens Healthcare, Erlangen, Germany). Susceptibility‐weighted images and T_1_‐weighted magnetization‐prepared 3D rapid gradient‐echo (MPRAGE) anatomical images were acquired. Parameters are identical to those previously described^5^ and are detailed in the supplementary methods. Follow‐up scans were performed after an average of 38.5 ± 4.4 months (mean ± SD).

### QSM Reconstruction

3D complex phase data (adaptively combined using scanner software) were unwrapped using a rapid path‐based minimum spanning tree algorithm (ROMEO).^19^ Brain masks were calculated from magnitude images using FSL's brain extraction tool (BET2).^20^ A noise map was estimated from inverted magnitude images and thresholded to allow noise‐based exclusion within the three outer voxel layers.^21^ Background field removal was completed using Laplacian boundary value extraction^22^ and 3D polynomial residual fitting.^23^ One voxel was eroded from the edge of the brain mask prior to dipole inversion, which was completed using multi‐scale dipole inversion (MSDI).^24^ All image processing was completed using MATLAB (The MathWorks, Inc., Natick, MA, USA) unless specified otherwise. Baseline and follow‐up susceptibility maps for all 89 subjects were visually inspected for quality control by a rater blind to clinical outcomes. After this, 8 subjects were excluded due to erroneous susceptibility maps (either at baseline or at follow‐up). This resulted in 8 PD participants being excluded from subsequent analyses.

### Spatial Standardization

For spatial standardization of susceptibility maps, we used a previously optimized methodology,^25^ adapted so that both baseline and follow‐up T1 images were used to produce the template. QSM images were brought into the template space using advanced normalization tools (ANTs, http://stnava.github.io/ANTs/) and visually inspected to ensure there were no errors in the applied transformation.

### Signed Versus Absolute QSM

Susceptibility maps generated using QSM contain both positive and negative values reflecting bulk paramagnetic and diamagnetic susceptibility, respectively (hereafter referred to as “signed” susceptibility). Use of *signed* susceptibility for whole‐brain analysis results in the averaging of positive and negative values in close proximity upon application of a smoothing kernel (required for voxel‐wise analysis), reducing statistical power. Therefore, we calculated the absolute values of susceptibility maps, equivalent to their magnitude (hereafter referred to as “absolute” susceptibility), for use in whole‐brain analysis. This removes information about para‐ and diamagnetic sources of susceptibility, but greatly increases statistical power for voxel‐wise analyses.^26^

### Whole‐Brain QSM Statistical Analysis

Voxel‐wise statistical analyses of QSM were performed using absolute QSM susceptibility. Images were spatially smoothed using a 3D Gaussian kernel (3‐mm standard deviation) and were subsequently smoothing compensated.^26^ Permutation analyses were performed with Randomise v2.9 and threshold‐free cluster enhancement (http://fsl.fmrib.ox.ac.uk/fsl/fslwiki/Randomise) in FSL. Significant clusters were inferred from a random subset of 10,000 data permutations and reported at family‐wise error‐corrected *P* < 0.05 (P_FWE_ < 0.05).

To investigate within‐group changes in susceptibility between visits, single‐group paired *t* tests adjusted for age, sex, and time between scans were carried out. To investigate how susceptibility related to motor and cognitive severity over time, regression analyses were performed for (1) baseline susceptibility against baseline MDS‐UPDRS‐III, and combined cognitive score (adjusted for age at baseline and sex); (2) baseline susceptibility against follow‐up MDS‐UPDRS‐III, and combined cognitive score (adjusted for age at baseline, sex, and time between scans); and (3) follow‐up susceptibility against follow‐up MDS‐UPDRS‐III and combined cognitive score (adjusted for age at follow‐up and sex). Given the primary interest in relating QSM to the clinical severity of PD, regression analyses were performed using only people with PD. However, they were additionally run in the control group to examine disease‐specific effects. We additionally ran exploratory analyses for follow‐up susceptibility versus follow‐up disease duration, MoCA, mood, REM Sleep Behavior Disorder Screening Questionnaire (RBDSQ), and the separate cognitive domain scores used to create the combined cognitive scores (word recognition, Hooper visual organization, Stroop color, verbal fluency letter, and verbal fluency category), adjusted for age at follow‐up and sex.

The QSM template and statistical maps were transformed into MNI152 space (Montreal Neurological Institute, McGill University, Canada) for display purposes.^25^

### Regional QSM Statistical Analysis

*Post‐hoc* region of interest (ROI) analyses were carried out to probe the nature of significant interactions observed in the voxel‐wise whole‐brain analysis between UPDRS‐III and combined cognitive score and *absolute* as well as *signed* susceptibility in the PD group, with the additional benefit of examining in more detail regions that are commonly implicated in PD. ROIs were chosen based on the follow‐up versus follow‐up results, as this is where we found the strongest associations. Use of *signed* susceptibility allowed us to determine whether significant voxel‐wise associations were likely due to diamagnetic or paramagnetic sources of susceptibility. Use of *absolute* susceptibility served as a point of comparison, and as confirmation for the whole‐brain results. The following ROIs were selected based on the voxel‐wise results: SN pars compacta (SNpc) and SN pars reticulata (SNpr), dentate nucleus, red nucleus, caudate nucleus, putamen, globus pallidus, hippocampus, nucleus basalis of Meynert (NBM), insular cortex, and lateral and medial orbitofrontal cortices. Details on how these anatomical regions were defined can be found in the supplementary methods, and they are shown in Figure S2. Unsmoothed, mean absolute, and signed susceptibility values were extracted from all ROIs and averaged across hemispheres to improve measurement stability. Before collapsing across hemispheres, interhemispheric differences were examined for all ROIs using *t* tests.

The associations between absolute and signed susceptibilities and motor and cognitive severity were examined using multiple linear regression, performed at each ROI for (1) baseline susceptibility versus baseline clinical severity (adjusted for age at baseline and sex), (2) baseline susceptibility versus follow‐up clinical severity (adjusted for age at baseline, sex, and time between scans), and (3) follow‐up susceptibility versus follow‐up clinical severity (adjusted for age at follow‐up and sex). Where significant associations were found in regions that displayed significantly different susceptibility between hemispheres, relationships in individual hemispheres were additionally examined. To facilitate a comparison with previous longitudinal QSM studies in PD, linear mixed models were fitted at each ROI to look at the change in *signed* susceptibility over time in PD (adjusted for age at baseline and sex). ANOVA tests were used to determine significance and relevant test‐statistics for each model, and *P*‐values were FDR adjusted across the 12 ROIs.^27^ All analyses were performed in R version 4.4.2, using the “lm” function for linear models, and the “lme4” package for linear mixed models (https://CRAN.R-project.org/package=lme4). Full model details including formulae are provided in the supplementary material.

### Voxel‐Based Morphometry

To compare the performance of QSM and conventional atrophy‐based measures in predicting clinical severity, whole‐brain voxel‐based morphometry (VBM) analyses mirroring the whole‐brain QSM analyses were also carried out. Segmentation, normalization to MNI152 space, and tissue probability modulation were carried out in SPM12 with default parameters, in conjunction with the DARTEL toolbox using a Gaussian smoothing kernel of 8‐mm full‐width‐at‐half‐maximum. Multiple regression models were implemented to examine associations between voxel‐wise gray and white matter volume and clinical parameters (combined cognitive score and UPDRS‐III). All models included age, sex, and total intracranial volume as nuisance covariates, and models examining baseline against follow‐up clinical scores were additionally adjusted for time between scans. Statistical parametric maps were generated for all models and reported at P_FWE_ < 0.05.

### Additional Statistical Analyses

To investigate whether the associations between susceptibility and combined cognitive sore and UPDRS‐III were driven by overall disease severity, we examined correlations at follow‐up between combined cognitive score, UPDRS‐III, and disease duration using Spearman's correlation coefficient.

## Results

### Association between Magnetic Susceptibility and Clinical Severity over Time

When interpreting these results, it should be noted that *lower* combined cognitive scores reflect poorer cognitive ability, whereas *higher* UPDRS‐III scores reflect poorer motor ability.

### Cognitive Severity

#### Voxel‐Wise Analysis

**FIG. 1:**
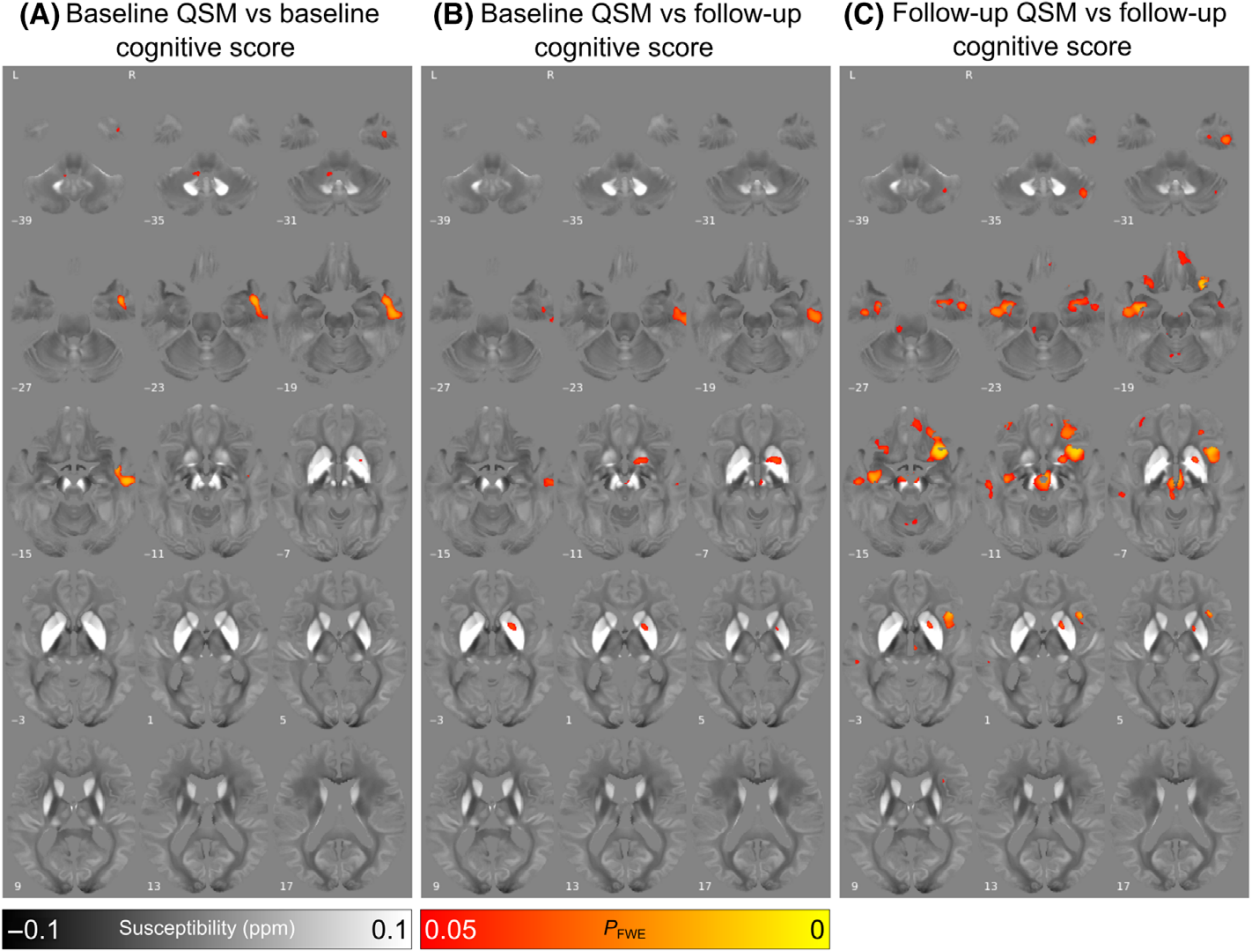
Relationship between increased absolute magnetic susceptibility and declining cognitive ability over the Parkinson's disease course, in a whole‐brain analysis. (**A**) Association between baseline susceptibility and combined cognitive score at baseline, adjusted for age at baseline and sex. (**B**) Association between baseline susceptibility and combined cognitive score at 3‐year follow‐up, adjusted for age at baseline, sex, and time between scans. Note the strong relationships between magnetic susceptibility and cognition in the basal forebrain. (**C**) Association between susceptibility at 36‐month follow‐up and combined cognitive score at 3‐year follow‐up, adjusted for age at follow‐up and sex. Note the additional relationships observed in temporal and frontal cortical regions. Results are overlaid on the study‐wise quantitative susceptibility mapping (QSM) template in MNI152 space, and numbers represent axial slice location in MNI152 space. Left side is shown on the left. Red/yellow clusters represent voxels where a significant relationship was seen at FWE‐corrected *P* < 0.05. [Color figure can be viewed at wileyonlinelibrary.com]

In people with PD, there were significant associations between increased baseline absolute susceptibility and decreased baseline combined cognitive score in the right temporal cortex and a small number of significant voxels in the right putamen (Fig. 1A, P_FWE_ < 0.05). We found a significant relationship between increased baseline absolute susceptibility and decreased follow‐up combined cognitive score in the right temporal cortex, basal forebrain, and putamen (Fig. 1B, P_FWE_ < 0.05). We found significant relationships between increased follow‐up absolute QSM and decreased follow‐up combined cognitive score, with additional involvement of bilateral temporal regions including hippocampi, brainstem regions including the red nucleus, as well as right and insular cortex (Fig. 1C, P_FWE_ < 0.05). In all three analyses the opposite contrasts (absolute QSM increasing with increasing combined cognitive score) revealed no significant clusters at P_FWE_ < 0.05.

We ran additional analyses to examine how susceptibility at follow‐up was related to individual components of the combined cognitive score at follow‐up. No significant associations were observed between susceptibility and MoCA, Hooper visual organization, word recognition, or verbal fluency letter (P_FWE_ < 0.05).

For both Stroop color and verbal fluency category, we observed significant clusters in the SN, red nucleus, NBM, anterior putamen, and regions of the thalamus, with increased susceptibility relating to poorer performance on those tasks (P_FWE_ < 0.05, Fig. S3). For Stroop color, we observed additional clusters in the dentate nucleus, right hippocampus, orbitofrontal and insular cortices. For verbal fluency category, we observed additional clusters in more widespread regions of the basal ganglia, including caudate nucleus and globus pallidus.

In the control group at the whole‐brain level, there were no significant associations between magnetic susceptibility and cognitive scores.

#### ROI Analysis

Post‐hoc ROI analyses of signed and absolute susceptibility mirroring the voxel‐wise analyses were carried out to examine the relative contribution of para‐ and diamagnetic susceptibility sources. At baseline (see Fig. S4), there was a significant negative association between cognition and absolute susceptibility in the lateral orbitofrontal cortex (P_FDR_ = 0.029, *β* = −2.2 × 10^−3^).

**FIG. 2:**
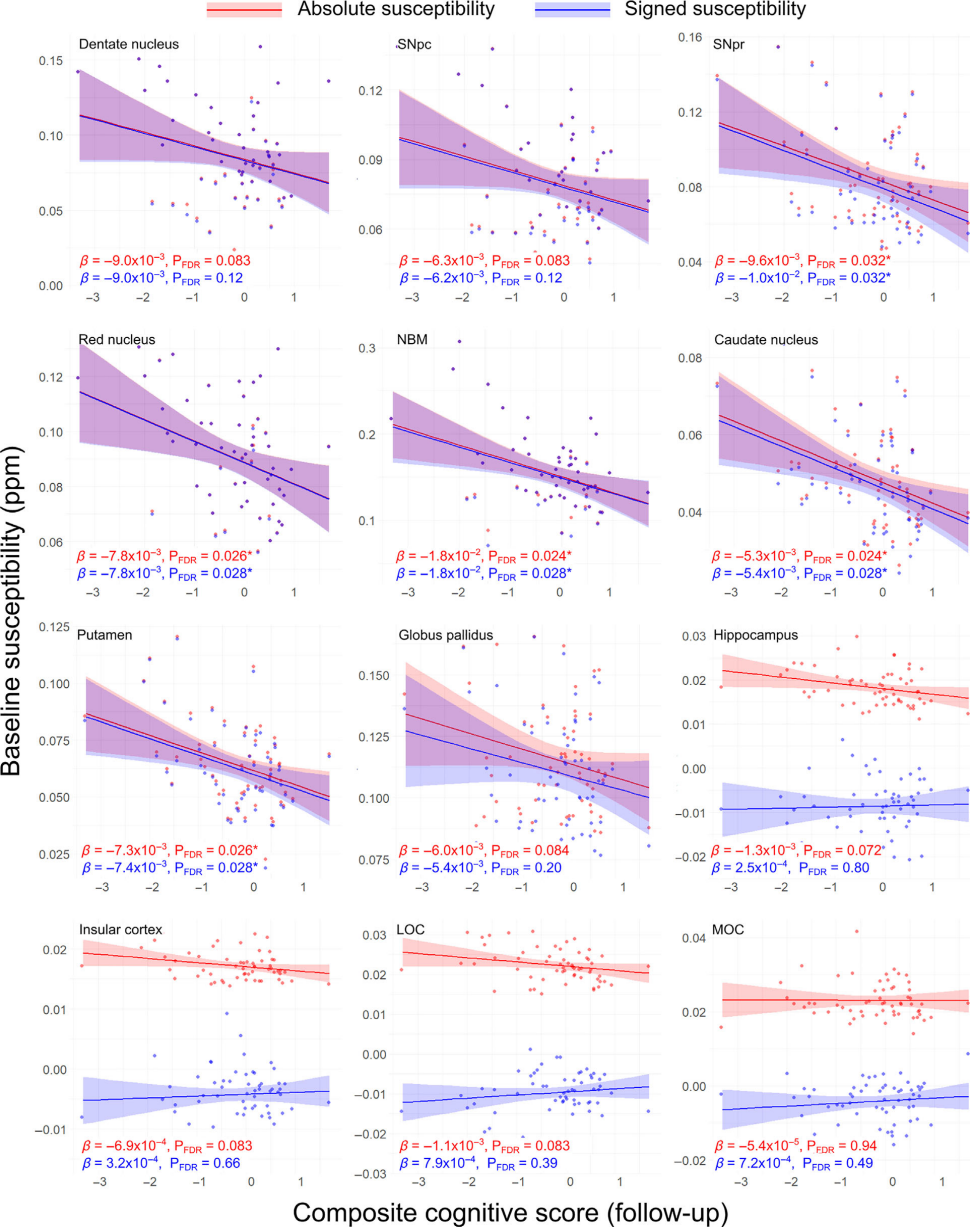
Regional relationships between baseline magnetic susceptibility and follow‐up cognitive score in Parkinson's disease. Data and statistics relating to regions of interest (ROI) mean absolute susceptibility are shown in red, and those relating to ROI mean signed susceptibility are shown in blue for 12 ROIs. Note that although relationships are essentially identical for absolute and signed quantitative susceptibility mapping (QSM) in the deep‐brain nuclei, they diverge in the cortical regions and hippocampus. Results are adjusted for age at baseline, sex, and time between scans. FDR‐corrected *P*‐values (P_FDR_) are presented, with asterisks indicating significant interactions at P_FDR_ < 0.05. *β* is the linear model coefficient associated with combined cognitive score. LOC, lateral orbitofrontal cortex; MOC, medial orbitofrontal cortex; NBM, nucleus basalis of Meynert; SNpc/pr, substantia nigra pars compacta/pars reticulata. [Color figure can be viewed at wileyonlinelibrary.com]

For the relationship between baseline susceptibility and follow‐up cognition (see Fig. 2), there were significant negative associations between follow‐up cognition and both absolute and signed susceptibilities in the SNpr (absolute/signed P_FDR_ = 0.032/0.032, *β* = −9.6 × 10^−3^/−1.0 × 10^−2^), red nucleus (P_FDR_ = 0.026/0.028, *β* = −7.8 × 10^−3^/−7.8 × 10^−3^), NBM (P_FDR_ = 0.024/0.028, *β* = −1.8 × 10^−2^/−1.8 × 10^−2^), caudate nucleus (P_FDR_ = 0.024/0.028, *β* = −5.4 × 10^−3^/−5.3 × 10^−3^), and putamen (P_FDR_ = 0.026/0.028, *β* = −7.3 × 10^−2^/−7.4 × 10^−2^). For subcortical regions, mean absolute and signed susceptibilities were almost identical. However, in cortical structures, notably, hippocampi, insular cortex, and lateral and medial orbitofrontal cortex, mean absolute and signed susceptibilities diverged, suggesting additional effects of diamagnetic sources in these regions on cognitive scores.

At follow‐up (see Fig. S5), there were significant negative associations between cognition and absolute and signed susceptibilities in the SNpc (absolute/signed P_FDR_ = 0.015/0.030, *β* = −9.2 × 10^−3^/−9.4 × 10^−3^), red nucleus (P_FDR_ = 0.0079/0.016, *β* = −1.0 × 10^−2^/−1.0 × 10^−2^), NBM (P_FDR_ = 0.015/0.030, *β* = −1.6 × 10^−2^/−1.6 × 10^−2^), and caudate nucleus (P_FDR_ = 0.015/0.030, *β* = −5.4 × 10^−3^/−5.5 × 10^−3^). In the SNpr there was a significant negative association between cognition and absolute susceptibility (P_FDR_ = 0.047, *β* = −8.3 × 10^−3^). In the cortical ROIs, there were significant positive associations observed between cognition and absolute susceptibility in the insular cortex (absolute/signed P_FDR_ = 0.0079/0.046, *β* = −1.2 × 10^−3^/1.4 × 10^−3^), and lateral orbitofrontal cortex (P_FDR_ = 0.015/0.030, *β* = −1.7 × 10^−3^/1.7 × 10^−3^). There were also significant negative associations between cognition and absolute susceptibility in the hippocampus (P_FDR_ = 0.015, *β* = −1.9 × 10^−3^), and medial occipital cortex (P_FDR_ = 0.015, *β* = −2.0 × 10^−3^). However, for signed QSM, there was a positive association with cognition seen in the insular and lateral orbitofrontal cortices. Full ROI statistics for the relationship between susceptibility and cognition can be seen in Table S1.

### Motor Severity

#### Voxel‐Wise Analysis

**FIG. 3:**
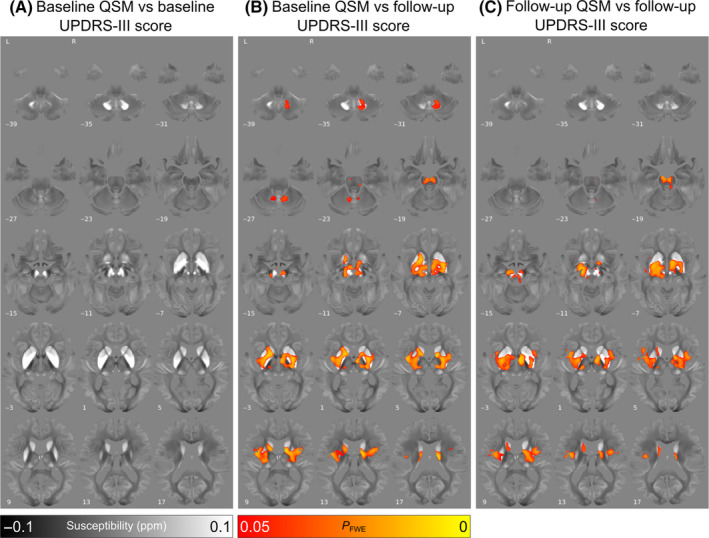
Relationship between increased absolute magnetic susceptibility and increasing motor severity over the Parkinson's disease course, in a whole‐brain analysis. (**A**) Association between baseline susceptibility and Movement Disorders Society Unified Parkinson's Disease Rating Scale Part III (MDS‐UPDRS III) score at baseline, adjusted for age at baseline and sex. (**B**) Association between baseline susceptibility and MDS‐UPDRS III score at 3‐year follow‐up, adjusted for age at baseline, sex, and time between scans. Note the strong relationships observed between magnetic susceptibility and motor severity in the basal ganglia and substantia nigra. (**C**) Association between susceptibility at 3‐year follow‐up and MDS‐UPDRS III score at 3‐year follow‐up, adjusted for age at follow‐up and sex. Results are overlaid on the study‐wise (QSM) template in MNI152 space, and numbers represent axial slice location in MNI152 space. Left side is shown on the left. Red/yellow clusters represent voxels where a significant relationship was seen at FWE‐corrected *P* < 0.05. [Color figure can be viewed at wileyonlinelibrary.com]

No significant clusters were found for increased baseline absolute susceptibility correlating with increased baseline MDS‐UPDRS III score (Fig. 3A). However, there were widespread regions found showing associations between increased baseline absolute susceptibility and increased follow‐up MDS‐UPDRS III score including bilaterally in the basal‐ganglia, SN, red nucleus, and insular cortex, as well as right dentate nucleus (Fig. 3B, P_FWE_ < 0.05). The relationship between increased follow‐up absolute susceptibility and increased follow‐up MDS‐UPDRS III score followed a similar pattern of regional involvement (Fig. 3C, P_FWE_ < 0.05). In all three analyses the opposite contrasts (absolute susceptibility increasing with decreasing MDS‐UPDRS III score) revealed no significant clusters at P_FWE_ < 0.05.

In the control group at the whole‐brain level, there were no significant associations between magnetic susceptibility and motor scores.

#### ROI Analysis

**FIG. 4:**
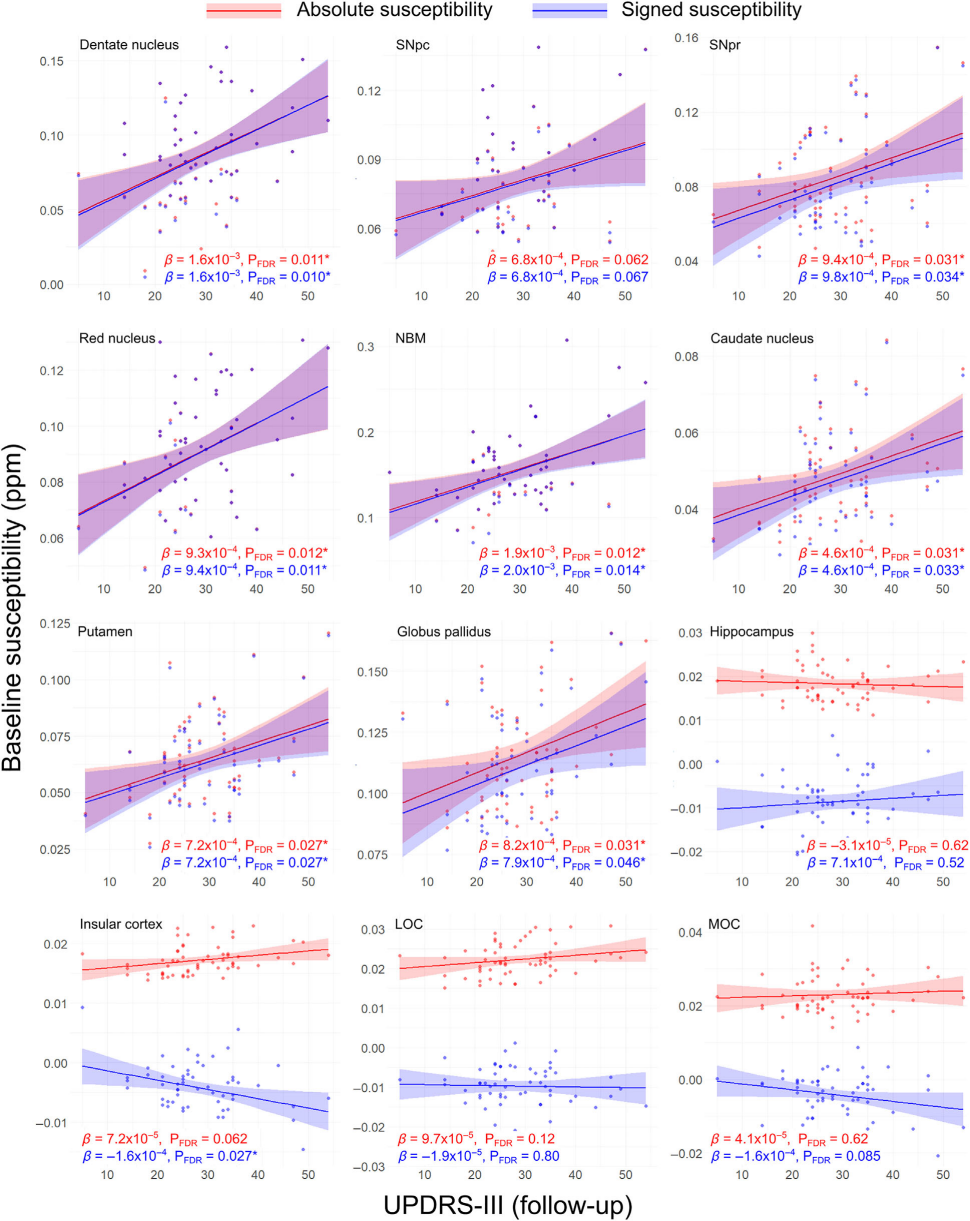
Regional relationships between baseline magnetic susceptibility and follow‐up motor severity score in PD. Data and statistics relating to absolute magnetic susceptibility are shown in red, and those relating to signed susceptibility are shown in blue for 12 regions of interest (ROI). Note that although relationships are essentially identical for absolute and signed quantitative susceptibility mapping (QSM) in the deep‐brain nuclei, they diverge in the cortical regions and hippocampus. Results are adjusted for age at baseline, sex, and time between scans. FDR‐corrected *P*‐values (P_FDR_) are presented, with asterisks indicating significant interactions at P_FDR_ < 0.05. *β* is the linear model coefficient associated with MDS‐UPDRS‐III score. LOC, lateral orbitofrontal cortex; MDS‐UPDRS‐III, Movement Disorders Society Unified Parkinson's Disease Rating Scale Part III; MOC, medial orbitofrontal cortex; NBM, nucleus basalis of Meynert; SNpc/pr, substantia nigra pars compacta/pars reticulata. [Color figure can be viewed at wileyonlinelibrary.com]

At ROI level, there were no significant relationships between baseline MDS‐UPDRS‐III score and baseline susceptibility (see Fig. S6). Looking at baseline susceptibility versus follow‐up MDS‐UPDRS III score (see Fig. 4), we observed significant positive associations between both absolute and signed susceptibility in the dentate nucleus (absolute/signed P_FDR_ = 0.011/0.010, *β* = 1.6 × 10^−3^/1.6 × 10^−3^), SNpr (P_FDR_ = 0.031/0.034, *β* = 9.4 × 10^−4^/9.8 × 10^−4^), red nucleus (P_FDR_ = 0.012/0.011, *β* = 9.3 × 10^−4^/9.4 × 10^−4^), NBM (P_FDR_ = 0.012/0.014, *β* = 1.9 × 10^−3^/2.0 × 10^−3^), caudate nucleus (P_FDR_ = 0.031/0.033, *β* = 4.6 × 10^−4^/4.7 × 10^−4^), putamen (P_FDR_ = 0.027/0.027, *β* = 7.2 × 10^−4^/7.2 × 10^−4^), and globus pallidus (P_FDR_ = 0.031/0.046, *β* = 8.2 × 10^−4^/7.9 × 10^−4^). Similar to the ROI analysis for QSM data and cognition, absolute and signed QSM data were almost overlapping for subcortical regions but showed differences in cortical regions. A significant negative association was found between baseline signed susceptibility and follow‐up MDS‐UPDRS III score in the insular cortex (P_FDR_ = 0.027, *β* = −1.6 × 10^−4^).

At follow‐up (see Fig. S7), there were significant positive associations between MDS‐UPDRS III score and both signed and absolute susceptibilities in the dentate nucleus (absolute/signed P_FDR_ = 0.013/0.014, *β* = 1.6 × 10^−3^/1.6 × 10^−3^), SNpr (P_FDR_ = 0.013/0.014, *β* = 1.2 × 10^−3^/1.3 × 10^−3^), NBM (P_FDR_ = 0.013/0.014, *β* = 1.8 × 10^−3^/1.8 × 10^−3^), caudate nucleus (P_FDR_ = 0.036/0.041, *β* = 4.7 × 10^−4^/4.8 × 10^−8^), and putamen (P_FDR_ = 0.031/0.040, *β* = 7.5 × 10^−4^/7.4 × 10^−4^). There was also a significant positive association of MDS‐UPDRS‐III score with absolute susceptibility in the globus pallidus (P_FDR_ = 0.036, *β* = 7.9 × 10^−4^). Again, we found opposite associations for absolute and signed QSM in cortical ROIs, with a positive association in the insular cortex at follow‐up between absolute susceptibility and MDS‐UPDRS III score (P_FDR_ = 0.036, *β* = 9.2 × 10^−5^), compared with a negative association with signed susceptibility (P_FDR_ = 0.041, *β* = −1.5 × 10^−4^). Full ROI statistics for motor severity can be seen in Table S2.

Only the globus pallidus displayed significantly different susceptibility between hemispheres, with the left side (0.120 ± 0.025, mean ± SD) having greater susceptibility than the right side (0.101 ± 0.025) at follow‐up, P_FDR_ = 0.0005, *T* = 4.25. We therefore examined the relationship in individual hemispheres between susceptibility in globus pallidus and motor severity in PD (Fig. S8). Both absolute and signed susceptibility in the left globus pallidus at follow‐up were significantly associated with follow‐up UPDRS‐III (absolute/signed, *P* = 0.008/0.009, *β* = 9.9 × 10^−4^/1.0 × 10^−3^), whereas neither signed nor absolute susceptibility in the right globus pallidus were (*P* = 0.069/0.140, *β* = 6.2 × 10^−4^/5.6 × 10^−3^).

### Other Disease Metrics

We found no signification associations between clinical measures of combined cognitive score, UPDRS‐III, and disease duration: cognition versus motor (*r* = −0.16, *P* = 0.23), cognition versus disease duration (*r* = −0.20, *P* = 0.13), motor versus disease duration (*r* = 0.21, *P* = 0.10).

The additional voxel‐wise analyses we carried to examine the association between follow‐up susceptibility and follow‐up markers of disease severity revealed no significant relationships at the whole‐brain level for RBDSQ, Hospital Anxiety and Depression Scale (HADS) depression, or HADS anxiety (P_FWE_ < 0.05). However, we did observe clusters indicating a positive relationship between disease duration and absolute susceptibility in the SN and red nucleus, (as well as a small cluster in the cerebellum) with a slight asymmetry in favor of the left hemisphere (P_FWE_ < 0.05, Fig. S9).

### Changes in Susceptibility over Time

#### Voxel‐Wise Analysis

In people with PD, absolute susceptibility significantly increased at follow‐up relative to baseline in the left precentral gyrus, left middle frontal cortex, and right middle temporal gyrus (P_FWE_ < 0.05, Fig. S10). In the control group, there were no significant clusters at whole brain indicating differences between baseline and follow‐up absolute susceptibility.

#### ROI Analysis

ROI analyses revealed decreasing signed susceptibility over time in PD in the dentate nucleus (*P* = 0.032, *β* = −5.7 × 10^−5^), red nucleus (*P* = 0.034, *β* = −7.4 × 10^−5^), and insular cortex (*P* = 0.015, *β* = −2.2 × 10^−5^), and increasing signed susceptibility in the medial orbitofrontal cortex (*P* = 0.045, *β* = 4.5 × 10^−5^). However, none of these effects remained significant after correction for multiple comparisons. Full ROI results for change in susceptibility over time in PD can be seen in Fig. S11 and Table S3.

### Voxel‐Based Morphometry

We did not find any significant associations between gray or white matter volume at baseline or follow‐up and clinical measures at baseline or follow‐up in the control or PD groups at P_FWE_ < 0.05.

## Discussion

In this longitudinal study, we have shown for the first time that magnetic susceptibility changes are predictive of future cognitive and motor outcomes in PD. Specifically, we showed higher baseline magnetic susceptibility in the right temporal cortex, right NBM, and right putamen relating to greater cognitive severity in PD after a mean follow‐up of 38 months, and higher baseline susceptibility in bilateral basal ganglia, SN, red nucleus, insular cortex, and dentate nucleus relating greater motor severity after 3‐year follow‐up. We further showed that this pattern of regional involvement persists at longitudinal follow‐up, with additionally increased absolute susceptibility in the hippocampus relating to increased cognitive severity. No such relationships were observed in relation to gray or white matter atrophy measured using voxel‐based morphometry.

We previously demonstrated that susceptibility relates to cognitive and motor severity in PD.^5^ This current study now extends those findings by showing that baseline susceptibility changes may predict later cognitive and motor severity in PD and that longitudinally, these associations become stronger, with more widespread regional involvement.

Although we found susceptibility increases, reflecting higher levels of brain tissue iron, predicting future cognitive and motor severity, we did not find susceptibility increases over time in the same regions. There have been only three longitudinal studies of QSM in PD,^14^, ^15^, ^16^ which taken together fail to show consistent increases in signed susceptibility values over time. Du et al^14^ examined 72 PD patients and 62 controls at baseline and after 18 months. Although they showed a relationship between change in signed susceptibility and change in UPDRS‐III in the SNpr, they did not find an increase in signed susceptibility in the SN over time, nor any relationship between change in signed susceptibility and MoCA scores.

Bergsland et al^15^ examined signed susceptibility values in the ventral posterior SN in 18 people with PD and 16 controls at baseline and 3‐year follow‐up. They found an increase in susceptibility in the ventral posterior SN in PD group but not controls. They did not find an association between baseline SN magnetic susceptibility and either baseline or follow‐up motor scores.

Guan et al^16^ examined longitudinal regional magnetic susceptibility in 38 PD patients with mean follow‐up of 16.8 months. They observed decreased signed tissue susceptibility in the SN, globus pallidus, and red nucleus after a mean of 16 months follow‐up, but no correlations with motor scores.

Consistent with these reports, we did not find robust regional increases in susceptibility over time, although we did find strong associations between magnetic susceptibility and measures of future clinical severity. This suggests that susceptibility may be somewhat limited in its ability to track, rather than predict, disease changes over time. The small number of longitudinal studies using QSM in PD may also reflect this lack of an effect, with a publication bias against negative findings.

Increases in tissue iron promote the production of toxic‐free reactive oxygen species, which in turn cause damage to DNA,^28^ affect mitochondrial function,^10^ and can modify proteins through reactive aldehydes,^29^ together leading to iron‐mediated cell death, or ferroptosis.^30^ In addition, excess iron can promote the aggregation of alpha synuclein fibrils.^11^ Our finding of higher susceptibilities in deep‐brain nuclei predicting poorer motor and cognitive outcomes is supportive of iron having a causative role in progressive neurodegeneration in PD. However, the lack of increase in regional susceptibility values over time suggests that this relationship is more complex than a gross increase in tissue iron over time.

Brain iron accumulates slowly over decades,^31^, ^32^, ^33^, ^34^, ^35^, ^36^ with some suggestion that the steepest increases are seen prior to 40 years of age, well before PD would be expected to manifest.^37^ It is possible that by the time PD clinically presents, much of the disease‐related iron accumulation has already occurred. Prodromal changes in proteins responsible for metal homeostasis could explain this, and areas with increased iron in PD have a higher intrinsic expression of genes relating to such processes.^13^

There is an apparent discrepancy between magnetic susceptibility being associated with future clinical severity and there being no susceptibility increases over time in the same regions. One possible reason to account for this is, while iron stored in ferritin macromolecules is the greatest contributor to brain tissue susceptibility,^38^, ^39^ there are other contributors. Metals including copper, magnesium, and calcium are diamagnetic, meaning they reduce overall magnetic susceptibility (relative to water or soft tissue).^40^ Myelin is also diamagnetic,^41^ and previous work has demonstrated increased cortical myelin production by oligodendrocytes in PD motor cortex, potentially due to changes in neuronal excitability.^42^ If this holds true for other regions, it could partially counteract the effect of cortical iron on susceptibility. Susceptibility can also be affected by changes in the orientation of tissue microstructure.^43^ Pathological proteins including tau, beta‐amyloid, and alpha‐synuclein tend to be diamagnetic as they contain many electron pairs,^44^ so they could also lower bulk susceptibility. The diamagnetic nature of tau and beta‐amyloid has been confirmed *in‐vitro* and in a mouse model of Alzheimer's disease (AD).^45^ Correlations between diamagnetic hippocampal susceptibility and tau and beta‐amyloid concentrations have also been demonstrated post‐mortem in human AD and primary age‐related tauopathy.^46^ Finally, tissue ferritin may become redistributed after cell death, drawing away major sources of tissue iron and lowering bulk susceptibility.

Separately examining absolute and signed susceptibility values may help disambiguate our failure to observe susceptibility increases over time in regions whose susceptibility is predictive of clinical severity. Although we were not able to use signed susceptibility in our voxel‐wise analyses, our ROI analyses using signed susceptibility corroborated the pattern seen at whole brain. In deep‐brain nuclei including the SN, basal ganglia, dentate nucleus, red nucleus, and NBM, ROI analyses using signed versus absolute susceptibilities produced nearly identical results. However, in hippocampal and cortical regions, the direction of the statistical relationships diverged, with *increased* absolute susceptibility but *decreased* signed susceptibility related to poorer cognitive and motor performance. This suggests that relationships in these regions include a contribution from diamagnetic susceptibility sources.

In the future, sequences sensitive to other tissue measures, such as multiparameter maps, with sensitivity to myelin, or alternative imaging with complementary information such as amyloid positron emission tomography computed tomography (PET‐CT), could be used alongside QSM to provide additional tissue information. Ultimately, combining in‐vivo, then ex‐vivo MRI with post‐mortem histology will enable disambiguation of changes on MRI with tissue composition, providing a more complete picture of neurodegenerative changes in PD dementia.

Interestingly, associations with susceptibility were observed in similar regions for both motor and cognitive severity scores. For example, susceptibility in regions such as the red nucleus (typically associated with motor symptoms) was associated with cognitive severity and, conversely, susceptibility in regions such as the NBM (typically associated with cognitive symptoms) was associated with motor severity. It therefore seems likely that findings for both motor and cognitive symptoms reflect, at least in part, a relationship between susceptibility and overall disease severity. The fact that there were no significant correlations between combined cognitive score, UPDRS‐III, and disease duration demonstrates that the results are not purely statistically driven. However, our voxel‐wise analysis comparing susceptibility and disease duration at follow‐up revealed significant clusters in the red nucleus and SN, which may suggest a common underlying neural substrate for cognitive and motor involvement in PD, particularly in these nuclei. It is also well known that the akinetic rigid motor subtype of PD is associated with poorer prognosis and increased risk of dementia compared to the tremor dominant subtype.^47^ Future work could examine the associations between susceptibility and axial features of PD.

Cortical regions showing significantly increased susceptibility relating to poorer cognition included orbitofrontal cortex (as well as other regions including hippocampi and insula), and not dorsolateral prefrontal cortex (DLPFC). DLPFC has been previously implicated in PD with mild cognitive impairment (PD‐MCI) and PD dementia (PDD), for example, from functional and structural brain imaging data,^48^ among other regions, including hippocampi and insula. However, other studies have additionally implicated the orbitofrontal cortex in PD‐MCI and PDD. For example, in a longitudinal study of cortical thinning in MCI,^49^ orbitofrontal thinning was found, as well as inferior parietal and occipital thinning. In another study of whole‐brain iron accumulation using QSM, increased QSM values were found in regions including orbitofrontal cortex (as well as cuneus, precuneus, and caudate) in patients with PD‐MCI compared with PD with normal cognition. Some of these discrepancies in brain regions may relate to heterogeneity in patient groups, as well as in a network of regions implicated in PD‐MCI and PDD, rather than single regions.^50^

Interestingly, the association between the combined cognitive score and susceptibility in the basal ganglia appeared to be more localized to the anterior putamen, which is known to connect with associative regions of the cortex.^51^

### Limitations

The current study was powered to detect susceptibility changes over time in people with PD, rather than differences between PD and control groups, and our lack of power likely reflects the relatively small control group in this longitudinal study.

Assessments of motor function were performed with participants in the ON state to reduce participant discomfort and anxiety, which could affect cognitive performance. Associations between susceptibility and motor function should therefore be interpreted with this in mind. So far, there have not been human studies showing that l‐dopa causally affects brain tissue iron content or magnetic susceptibility, and previous work has shown that l‐dopa does not alter cortical iron levels in a mouse model of PD.^52^ In humans, magnetic susceptibility in subcortical regions does correlate with l‐dopa dose,^53^ although this is likely to reflect an association with disease severity. As l‐dopa would be expected to mask severity of motor symptoms, the actual relationship between QSM and motor severity may even be stronger than the one reported in this paper. Future work could specifically examine the strength of association between motor function and susceptibility in patients both ON and OFF l‐dopa.

Our findings so far can only relate to PD patients examined as a group. Ultimately, imaging changes providing information on an individual level will be needed to provide prognostic information to individuals in the clinic.

## Conclusions

We have shown that magnetic susceptibility changes in relevant brain regions are predictive of cognitive and motor severity after 3‐year follow‐up, with increased susceptibility in the NBM related to poorer future cognitive severity, and increased magnetic susceptibility in the basal ganglia related to poorer future motor severity. However, susceptibility increases in these regions were not found over time. Future work should combine imaging modalities sensitive to myelin and pathological protein accumulation to augment these findings and elucidate how they relate to tissue composition changes and measures of clinical severity in PD and PD dementia.

## Author Roles

(1) Research project: A. Conception, B. Organization, C. Execution; (2) Statistical Analysis: A. Design, B. Execution, C. Review and Critique; (3) Manuscript: A. Writing of the First Draft, B. Generation of Figures, C. Review and Critique.

G.E.C.T.: 1A, 2A, 2B, 2C, 3A, 3B, 3C

N.H.: 1B, 1C, 2C, 3C

A.Z.: 1B, 1C, 2C, 3C

K.S.: 2A, 2C, 3C

R.S.W.: 1A, 1B, 1C, 2A, 2C, 3C

## Supporting information


**Data S1.** Supporting information.

## Data Availability

Anonymised, group‐level summary QSM data and code for performing group‐level analyses are available on github: https://github.com/gecthomas/QSM_PD_longitudinal.
