# Supplementary material for: Longitudinal Associations of Magnetic Susceptibility with Clinical Severity in Parkinson's Disease
Source: Mov Disord. 2024 Jan 3;39(3):546–59. doi: 10.1002/mds.29702 (PMC11141787; doi:10.1002/mds.29702)
Supplement: Supplementary file 1 — Data S1. Supporting information. [file MDS-39-546-s001.pdf]

Supplementary material

Longitudinal associations of magnetic susceptibility with clinical severity in Parkinson's disease

George EC Thomas[1]*, Naomi Hannaway[1], Angelika Zarkali[1], Karin Shmueli[2] & Rimona S Weil[1,3,4]

1. Dementia Research Centre, UCL Institute of Neurology, London, UK
2. Department of Medical Physics and Biomedical Engineering, UCL, London, UK
3. Wellcome Centre for Human Neuroimaging, UCL, London, UK
4. Movement Disorders Consortium, UCL, London, UK

Supplementary methods

Supplementary tables 1-3

Supplementary figures 1-11

Supplementary methods

MRI acquisition parameters

Images were acquired on a Siemens Prisma-fit 3T MRI system using a 64-channel receive array coil (Siemens Healthcare, Erlangen, Germany) with parameters identical to those previously described[1]. Susceptibility-weighted MRI signals were obtained from a 2×1-accelerated[2], 3D flow-compensated spoiled-gradient-recalled echo sequence: flip angle=12°; echo time=18ms; repetition time=25ms; receiver bandwidth=110Hz/pixel; matrix size=204×224×160 with 1×1×1mm^3 voxel size; scan time=5min41s. T1-weighted magnetization-prepared 3D rapid gradient-echo (MPRAGE) anatomical images were also acquired: 2×1 parallel acceleration; inversion time=1100ms; flip angle=7°; first echo time=3.34ms; echo spacing=7.4ms; repetition time=2530ms; receiver bandwidth=200Hz/pixel; matrix dimensions=256×256×176; voxel size=1×1×1mm^3; scan time=6min3s.

Definition of anatomical regions for ROI analysis

The caudate nucleus, globus pallidus, putamen and hippocampus were automatically segmented from the study-wise MPRAGE template using FSL-FIRST. The red nucleus and dentate nucleus were segmented from the MPRAGE and QSM templates using MRIcloud[3, 4]. The SNpc and SNpr were manually traced on the MPRAGE template, as described previously[5]. The NBM was manually traced as described previously[1]. All subcortical regions of interest were eroded by convolution with a 1-mm radius spherical kernel to minimise partial-volume effects. The insula, lateral and medial orbitofrontal cortices were defined using the Desikan-Killiany-Tourville digital atlas. The OASIS-30 template and OASIS-TRT20 joint fusion atlas were obtained from Mindboggle (mindboggle.info/data.html). The study-wise-template to OASIS-30 space non-linear transformations were calculated as described previously[5]. To reduce partial-volume contamination, each cortical ROI was intersected with a study-wise average grey matter mask (generated in SPM12, https://www.fil.ion.ucl.ac.uk/spm/software/spm12/) binarized at a probability density cut-off of 0.5.

Definition of linear and linear mixed models for ROI analysis

The relationships between susceptibility and clinical severity at baseline and follow-up were examined by fitting linear models at each ROI as follows: $QSM(ROI)_{ik} = \beta_0 +$

$\beta_1 Age_{ik} + \beta_2 Sex_i + \beta_3 clin_{ik} + \varepsilon_{ik}$, where $QSM(ROI)_{ik}$ is the regional mean absolute or signed susceptibility at timepoint k for subject i, $clin_{ik}$ is either the combined cognitive or MDS-UPDRS-III score for participant i at timepoint k, Age_{ik} is the age of participant i at timepoint k, and Sex_i is the sex of participant i. In all linear and linear mixed models, β and ε are the model coefficients and fit residuals, respectively. To account for multiple comparisons, p-values were FDR adjusted across the 12 ROIs[6]. The relationship between susceptibility and future motor and cognitive severity was tested as follows: $QSM(ROI)_{ik1} = \beta_0 + \beta_1 Age_{ik1} + \beta_2 Sex_i + \beta_3 Tfup_i + \beta_4 clin_{ik2} + \varepsilon_i$, where $QSM(ROI)_{ik1}$ is the regional mean absolute or signed susceptibility at baseline for participant i, $clin_{ik2}$ is either the combined cognitive or MDS-UPDRS-III score for participant i at follow-up, and Age_{ik1}, $Tfup_i$ and Sex_i are the baseline age, time between scans and sex for participant i, respectively. ANOVA tests were used to determine significance and relevant test-statistics for each model.

To facilitate comparisons to previous longitudinal QSM studies in PD, a set of ROI analyses to look at the change in the *signed* susceptibilities in PD over time were carried out. To do this, the following linear mixed model was fitted at each ROI: $QSM(ROI)_{ik} = \beta_0 + \beta_1 Age_{ik1} + \beta_2 Sex_i + \beta_3 Tfup_{ik} + b_{0i} + \varepsilon_{ik}$, where $QSM(ROI)_{ik}$ is the regional mean signed susceptibility at timepoint k for participant i, $Tfup_{ik}$ is the time from baseline at timepoint k for participant i (this will be zero at baseline), Age_{ik1} and Sex_i are the age at baseline and sex for participant i, and b_{0i} is a random intercept effect per subject.

	Baseline susceptibility vs baseline combined cognitive score			Baseline susceptibility vs follow-up combined cognitive score			Follow-up susceptibility vs follow-up combined cognitive score		
	**β**	**P**	**P_{FDR}**	**β**	**P**	**P_{FDR}**	**β**	**P**	**P_{FDR}**
Absolute susceptibility									
Dentate nucl.	-9.4e-03	0.12	0.21	-9.0e-03	0.063	0.083	-9.7e-03	0.054	0.065
SNpc	-4.7e-03	0.27	0.30	-6.3e-03	0.056	0.083	***-9.2e-03***	***0.0098***	***0.015***
SNpr	-6.0e-03	0.21	0.29	***-9.6e-03***	***0.013***	***0.032***	***-8.3e-03***	***0.035***	***0.047***
Red nucleus	-9.0e-03	0.023	0.068	***-7.8e-03***	***0.0088***	***0.026***	***-1.0e-02***	***0.0013***	***0.0079***
NBM	-1.0e-02	0.20	0.29	***-1.8e-02***	***0.0039***	***0.024***	***-1.6e-02***	***0.0082***	***0.015***
Caudate nucl.	-5.4e-03	0.019	0.068	***-5.3e-03***	***0.0038***	***0.024***	***-5.4e-03***	***0.0061***	***0.015***
Putamen	-6.5e-03	0.055	0.13	***-7.3e-03***	***0.0067***	***0.026***	-5.1e-03	0.080	0.087
Globus pallidus	-4.3e-03	0.30	0.30	-6.0e-03	0.077	0.084	-3.4e-03	0.32	0.32
Hippocampus	-1.4e-03	0.074	0.15	-1.3e-03	0.036	0.072	***-1.9e-03***	***0.0052***	***0.015***
Insular cortex	-1.1e-03	0.014	0.068	-6.9e-04	0.050	0.083	***-1.2e-03***	***0.0019***	***0.0079***
LOC	***-2.2e-03***	***0.0024***	***0.029***	-1.1e-03	0.069	0.083	***-1.7e-03***	***0.0056***	***0.015***
MOC	-1.0e-03	0.28	0.30	-5.4e-05	0.94	0.94	***-2.0e-03***	***0.0087***	***0.015***
Signed susceptibility									
Dentate nucl.	-9.4e-03	0.13	0.31	-9.0e-03	0.068	0.12	-9.7e-03	0.061	0.082
SNpc	-4.6e-03	0.29	0.44	-6.2e-03	0.065	0.12	***-9.4e-03***	***0.011***	***0.030***
SNpr	-6.0e-03	0.25	0.44	***-1.0e-02***	***0.013***	***0.032***	*-8.7e-03*	*0.041*	*0.065*
Red nucleus	-8.9e-03	0.024	0.14	***-7.8e-03***	***0.0092***	***0.028***	***-1.0e-02***	***0.0014***	***0.016***
NBM	-9.0e-03	0.29	0.44	***-1.8e-02***	***0.0076***	***0.028***	***-1.6e-02***	***0.013***	***0.030***
Caudate nucl.	-5.4e-03	0.023	0.14	***-5.4e-03***	***0.0044***	***0.028***	***-5.5e-03***	***0.0066***	***0.030***
Putamen	-6.4e-03	0.062	0.19	***-7.4e-03***	***0.0068***	***0.028***	-5.3e-03	0.074	0.089
Globus pallidus	-3.0e-03	0.50	0.55	-5.4e-03	0.134	0.20	-3.1e-03	0.39	0.39
Hippocampus	-8.1e-04	0.50	0.55	2.5e-04	0.80	0.80	2.0e-03	0.043	0.065
Insular cortex	-7.5e-05	0.93	0.93	3.2e-04	0.61	0.66	***1.4e-03***	***0.023***	***0.046***
LOC	1.7e-03	0.059	0.19	7.9e-04	0.30	0.39	***1.7e-03***	***0.012***	***0.030***
MOC	8.2e-04	0.46	0.55	7.2e-04	0.40	0.49	1.0e-03	0.34	0.36

Supplementary Table 1 – Regional associations between signed and absolute susceptibility and cognitive ability assessed using a combined cognitive score. Baseline vs baseline results are adjusted for age at baseline and sex. Baseline vs follow-up results are adjusted for age at baseline, sex and time between scans. Follow-up vs follow-up results are adjusted for age at follow-up and sex. Uncorrected and FDR-corrected p-values are presented, with bold typeface indicating significant associations. β is the linear model coefficient associated with combined cognitive score. SNpc/pr = substantia nigra pars compacta / pars reticulata; NBM = nucleus basalis of Meynert; MOC = medial orbitofrontal cortex; LOC = lateral orbitofrontal cortex.

	Baseline susceptibility vs baseline UPDRS-III			Baseline susceptibility vs follow-up UPDRS-III			Follow-up susceptibility vs follow-up UPDRS-III		
	**β**	**P**	**P_{FDR}**	**β**	**P**	**P_{FDR}**	**β**	**P**	**P_{FDR}**
Absolute susceptibility									
Dentate nucl.	3.0e-04	0.41	0.61	***1.6e-03***	***8.8e-4***	***0.011***	***1.6e-03***	***0.0018***	***0.013***
SNpc	3.5e-04	0.15	0.55	*6.8e-04*	*0.044*	*0.062*	6.4e-04	0.084	0.11
SNpr	5.0e-04	0.074	0.45	***9.4e-04***	***0.018***	***0.031***	***1.2e-03***	***0.0022***	***0.013***
Red nucleus	2.3e-04	0.32	0.55	***9.3e-04***	***0.0020***	***0.012***	*6.9e-04*	*0.042*	*0.063*
NBM	8.3e-04	0.074	0.45	***1.9e-03***	***0.0029***	***0.012***	***1.8e-03***	***0.0046***	***0.019***
Caudate nucl.	1.5e-04	0.25	0.55	***4.6e-04***	***0.015***	***0.031***	***4.7e-04***	***0.020***	***0.036***
Putamen	9.9e-05	0.62	0.72	***7.2e-04***	***0.0088***	***0.027***	***7.5e-04***	***0.010***	***0.031***
Globus pallidus	2.8e-04	0.25	0.55	***8.2e-04***	***0.016***	***0.031***	***7.9e-04***	***0.021***	***0.036***
Hippocampus	2.0e-05	0.66	0.72	-3.1e-05	0.62	0.62	1.2e-04	0.11	0.13
Insular cortex	-1.5e-05	0.57	0.72	*7.2e-05*	*0.047*	*0.062*	***9.2e-05***	***0.019***	***0.036***
LOC	-9.8e-06	0.82	0.82	9.7e-05	0.10	0.12	4.3e-05	0.50	0.54
MOC	-5.7e-05	0.30	0.55	4.1e-05	0.60	0.62	3.6e-05	0.65	0.65
Signed susceptibility									
Dentate nucl.	3.2e-04	0.38	0.51	***1.6e-03***	***8.2e-4***	***0.010***	***1.6e-03***	***0.0017***	***0.014***
SNpc	3.6e-04	0.15	0.51	6.8e-04	0.050	0.067	6.4e-04	0.094	0.13
SNpr	5.4e-04	0.073	0.44	***9.8e-04***	***0.021***	***0.034***	***1.3e-03***	***0.0023***	***0.014***
Red nucleus	2.4e-04	0.31	0.51	***9.4e-04***	***0.0018***	***0.011***	*7.0e-04*	*0.042*	*0.063*
NBM	9.0e-04	0.065	0.44	***2.0e-03***	***0.0034***	***0.014***	***1.8e-03***	***0.0055***	***0.022***
Caudate nucl.	1.5e-04	0.27	0.51	***4.7e-04***	***0.017***	***0.033***	***4.8e-04***	***0.020***	***0.041***
Putamen	1.1e-04	0.60	0.66	***7.2e-04***	***0.0096***	***0.027***	***7.4e-04***	***0.013***	***0.040***
Globus pallidus	2.4e-04	0.35	0.51	***7.9e-04***	***0.031***	***0.046***	*7.7e-04*	*0.036*	*0.061*
Hippocampus	-8.1e-05	0.25	0.51	7.1e-05	0.48	0.52	-1.4e-04	0.17	0.21
Insular cortex	-4.6e-05	0.33	0.51	***-1.6e-04***	***0.011***	***0.027***	***-1.5e-04***	***0.021***	***0.041***
LOC	6.2e-07	0.99	0.99	-1.9e-05	0.80	0.80	-1.3e-05	0.86	0.86
MOC	3.4e-05	0.58	0.66	-1.6e-04	0.071	0.085	-3.4e-05	0.76	0.83

Supplementary Table 2 – Regional associations between signed and absolute susceptibility and motor ability assessed with the UPDRS-III score. Baseline vs baseline results are adjusted for age at baseline and sex. Baseline vs follow-up results are adjusted for age at baseline, sex and time between scans. Follow-up vs follow-up results are adjusted for age at follow-up and sex. Uncorrected and FDR-corrected p-values are presented, with bold typeface indicating significant associations. β is the linear model coefficient associated with UPDRS-III score. SNpc/pr = substantia nigra pars compacta / pars reticulata; NBM = nucleus basalis of Meynert; MOC = medial orbitofrontal cortex; LOC = lateral orbitofrontal cortex.

	Marginal R^2	Conditional R^2	β	P	P_{FDR}
Dentate nucleus	0.064	0.97	$-5.7x10^{-5}$	0.033	0.13
SNpc	0.017	0.89	$5.3\ x10^{-5}$	0.16	0.27
SNpr	0.036	0.90	$-1.8x10^{-5}$	0.68	0.73
Red nucleus	0.022	0.89	$-7.4x10^{-5}$	0.034	0.13
NBM	0.16	0.93	$5.8x10^{-5}$	0.33	0.50
Caudate nucleus	0.084	0.93	$3.0x10^{-5}$	0.081	0.20
Putamen	0.18	0.92	$2.3x10^{-5}$	0.41	0.55
Globus pallidus	0.027	0.87	$-1.8x10^{-5}$	0.66	0.72
Hippocampus	0.036	0.68	$-2.7x10^{-5}$	0.13	0.25
Insula	0.16	0.82	$-2.2x10^{-5}$	0.015	0.13
LOC	0.15	0.75	$-4.2x10^{-6}$	0.73	0.73
MOC	0.044	0.47	$4.5x10^{-5}$	0.045	0.13

Supplementary Table 3 – Results of linear mixed modelling showing regional change in signed magnetic susceptibility over time in Parkinson's disease. Change in susceptibility is modelled by fixed effects for time to follow-up (months), age at baseline and sex, and a random intercept effect per subject. The marginal R^2 indicates the variance explained by fixed effects only, while the conditional R^2 indicates variance explained by both fixed and random effects. B is the coefficient of the fixed effect for time to follow-up on susceptibility, positive values indicate increasing susceptibility over time and negative values indicate decreasing susceptibility over time. Uncorrected P values indicate the significance of the effect of follow-up time on susceptibility, adjusted for age at baseline, sex and subject. P_{FDR} values are corrected across multiple comparisons. SNpc/pr = substantia nigra pars compacta / pars reticulata; NBM = nucleus basalis of Meynert; MOC = medial orbitofrontal cortex; LOC = lateral orbitofrontal cortex.

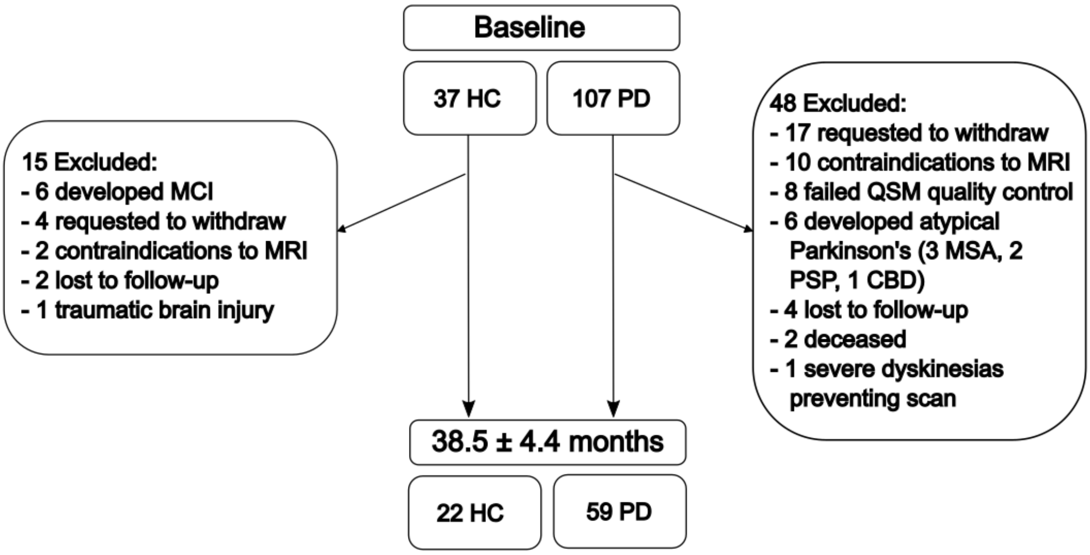

Supplementary Figure 1 – Flowchart indicating participants involved in the study. Parkinson's disease patients and controls tested in the study at baseline and after 36-month follow-up. Reasons for exclusion and data loss between baseline and follow-up visits are indicated. CBD = corticobasal degeneration; MSA = multiple system atrophy; PSP = progressive supranuclear palsy; QSM = quantitative susceptibility mapping.

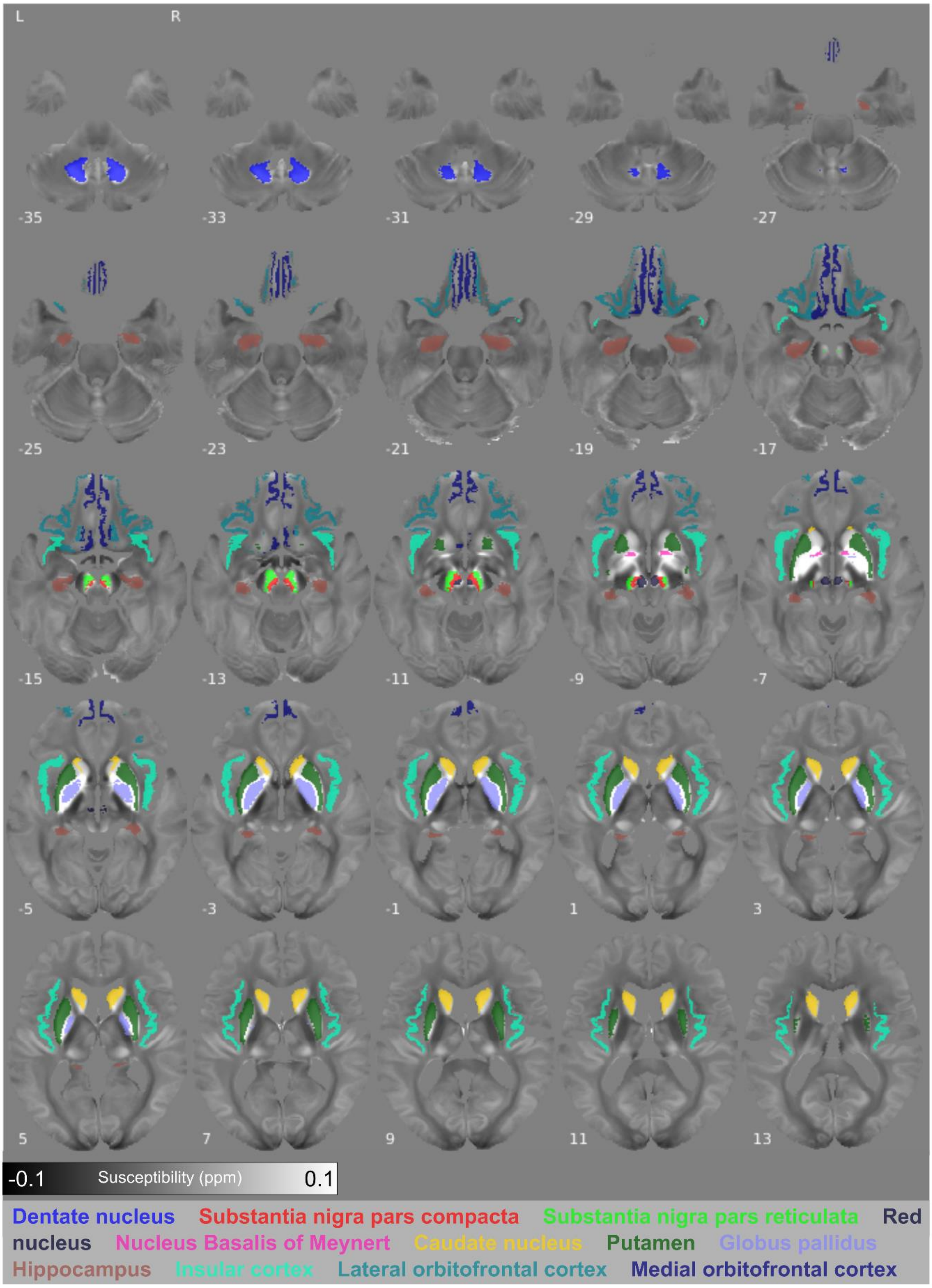

Supplementary Figure 2 – Regions of interest visualised on the study-wise QSM template in MNI space. Regions are indicated by the colour coded text in the bottom panel. Numbers indicate axial slice position in MNI space.

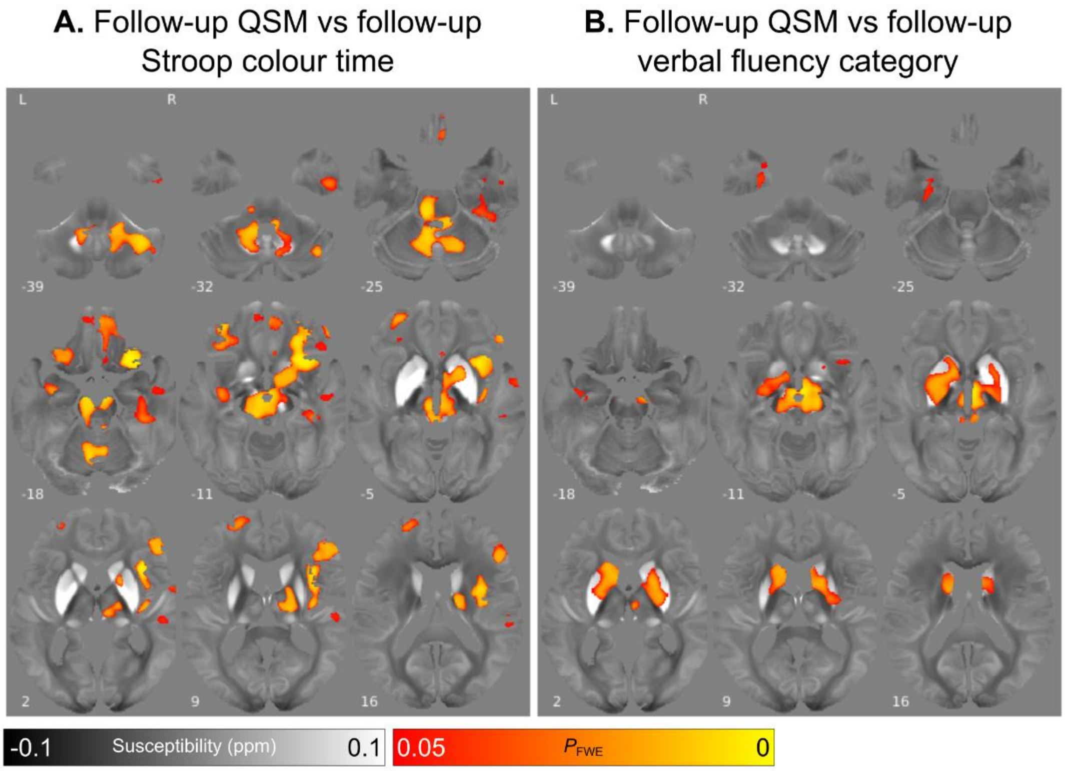

Supplementary Figure 3 – Relationship between follow-up absolute magnetic susceptibility and follow-up cognitive domain scores, in a whole brain analysis. A. Positive association between follow-up susceptibility and follow-up Stroop colour time (greater time taken meaning poorer performance), adjusted for age at baseline and sex. **B.** Negative association between follow-up susceptibility follow-up verbal fluency category score (lower scores meaning poorer performance), adjusted for age at baseline and sex. Red/yellow clusters represent voxels where a significant relationship was seen at FWE-corrected P<0.05. Results are overlaid on the study-wise QSM template in MNI152 space, and numbers represent axial slice location in MNI152 space.

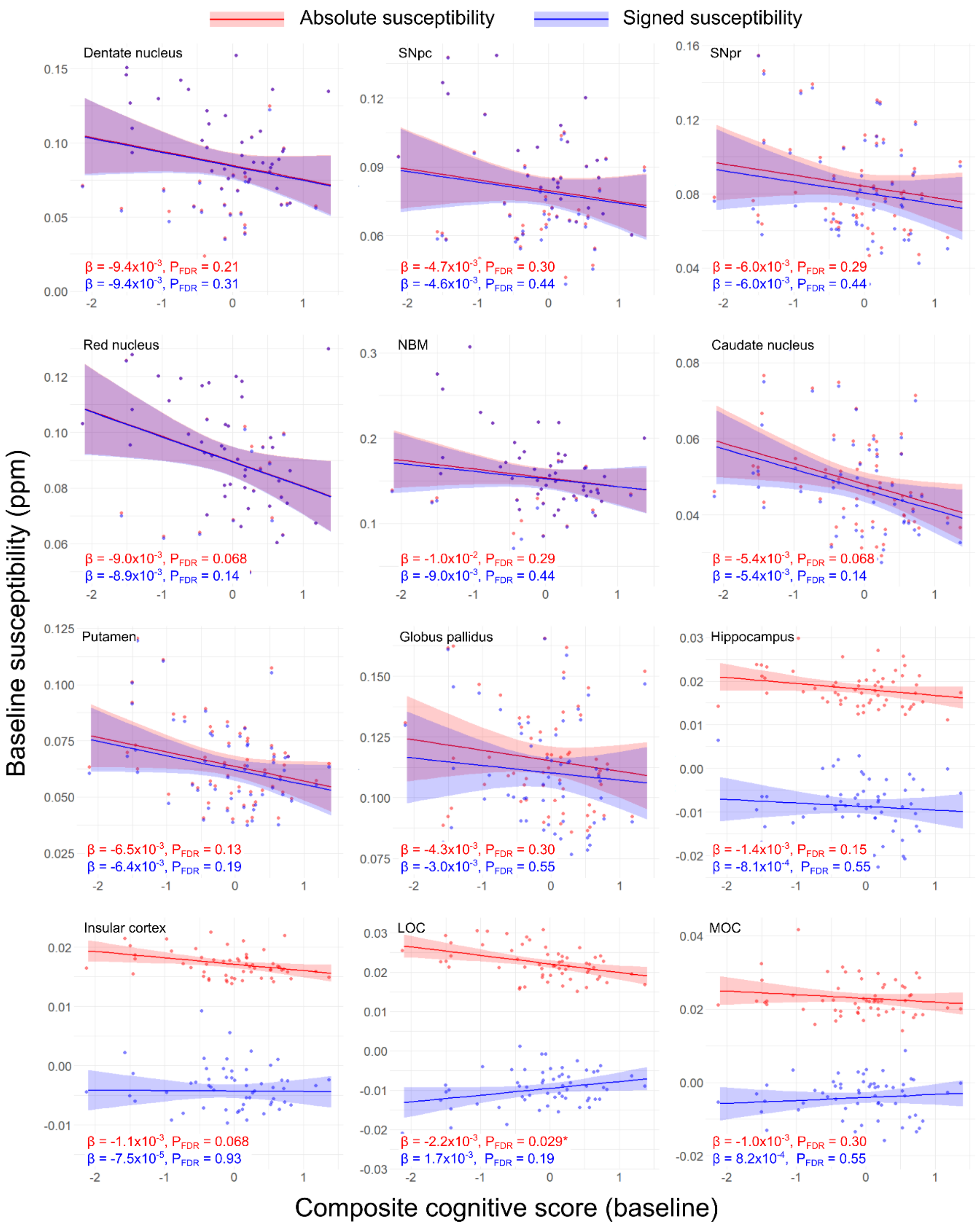

Supplementary Figure 4 – Regional relationships between baseline magnetic susceptibility and baseline cognitive score in Parkinson's disease. Data and statistics relating to ROI mean absolute susceptibility are shown in red, and those relating to ROI mean signed susceptibility are shown in blue. Results are adjusted for age at baseline and sex. FDR-corrected p-values (P_{FDR}) are presented, with asterisks indicating significant interactions at $P_{FDR} < 0.05$. β is the linear model coefficient associated with combined cognitive score. SNpc/pr = substantia nigra pars compacta / pars reticulata; NBM = nucleus basalis of Meynert; MOC = medial orbitofrontal cortex; LOC = lateral orbitofrontal cortex.

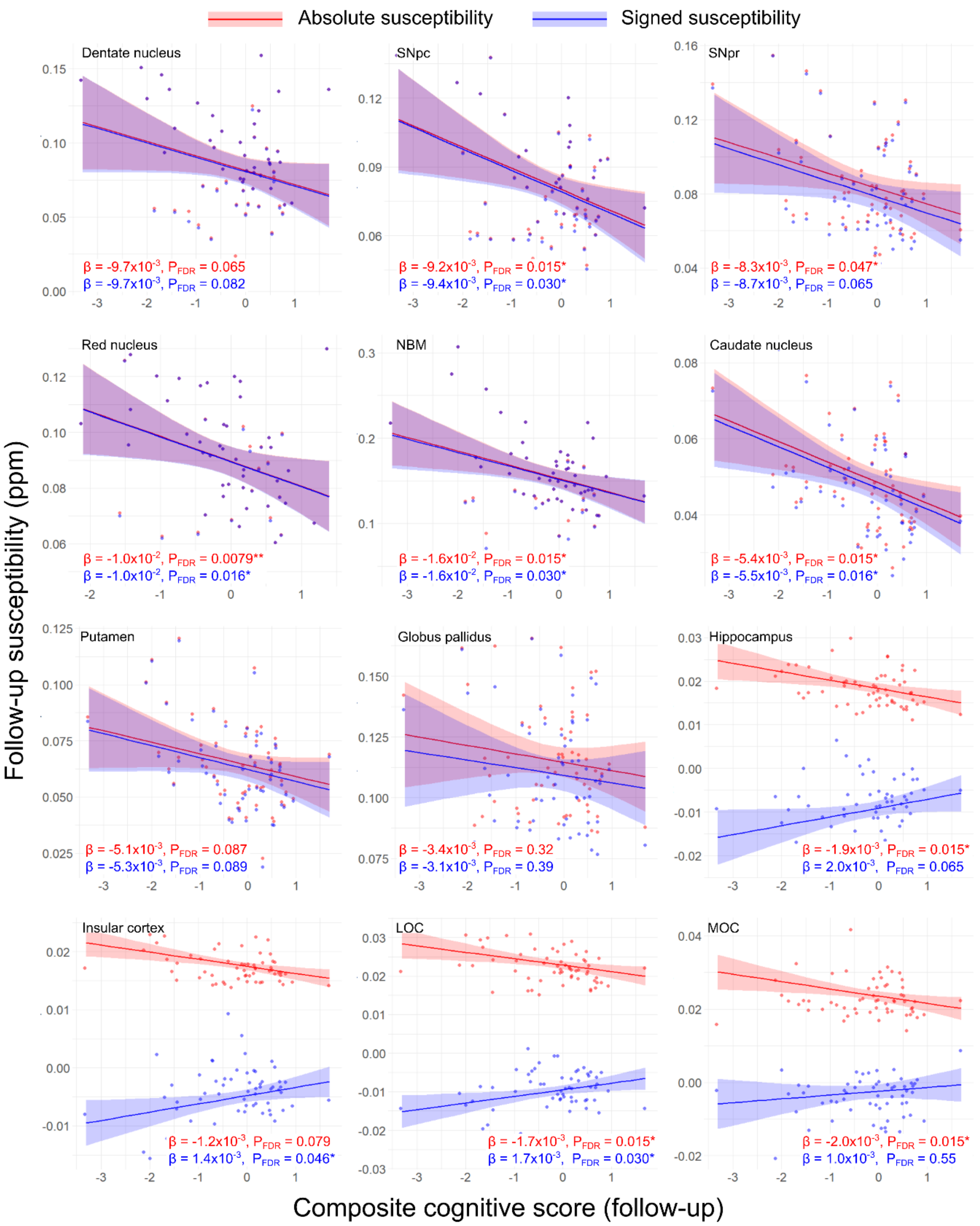

Supplementary Figure 5 – Regional relationships between follow-up magnetic susceptibility and follow-up cognitive score in Parkinson's disease. Data and statistics relating to ROI mean absolute susceptibility are shown in red, and those relating to ROI mean signed susceptibility are shown in blue. Results are adjusted for age at follow-up and sex. FDR-corrected p-values (P_{FDR}) are presented, with single asterisks indicating significant interactions at $P_{FDR} < 0.05$, and double asterisks indicating $P_{FDR} < 0.01$. β is the linear model coefficient associated with combined cognitive score. SNpc/pr = substantia nigra pars compacta / pars reticulata; NBM = nucleus basalis of Meynert; MOC = medial orbitofrontal cortex; LOC = lateral orbitofrontal cortex.

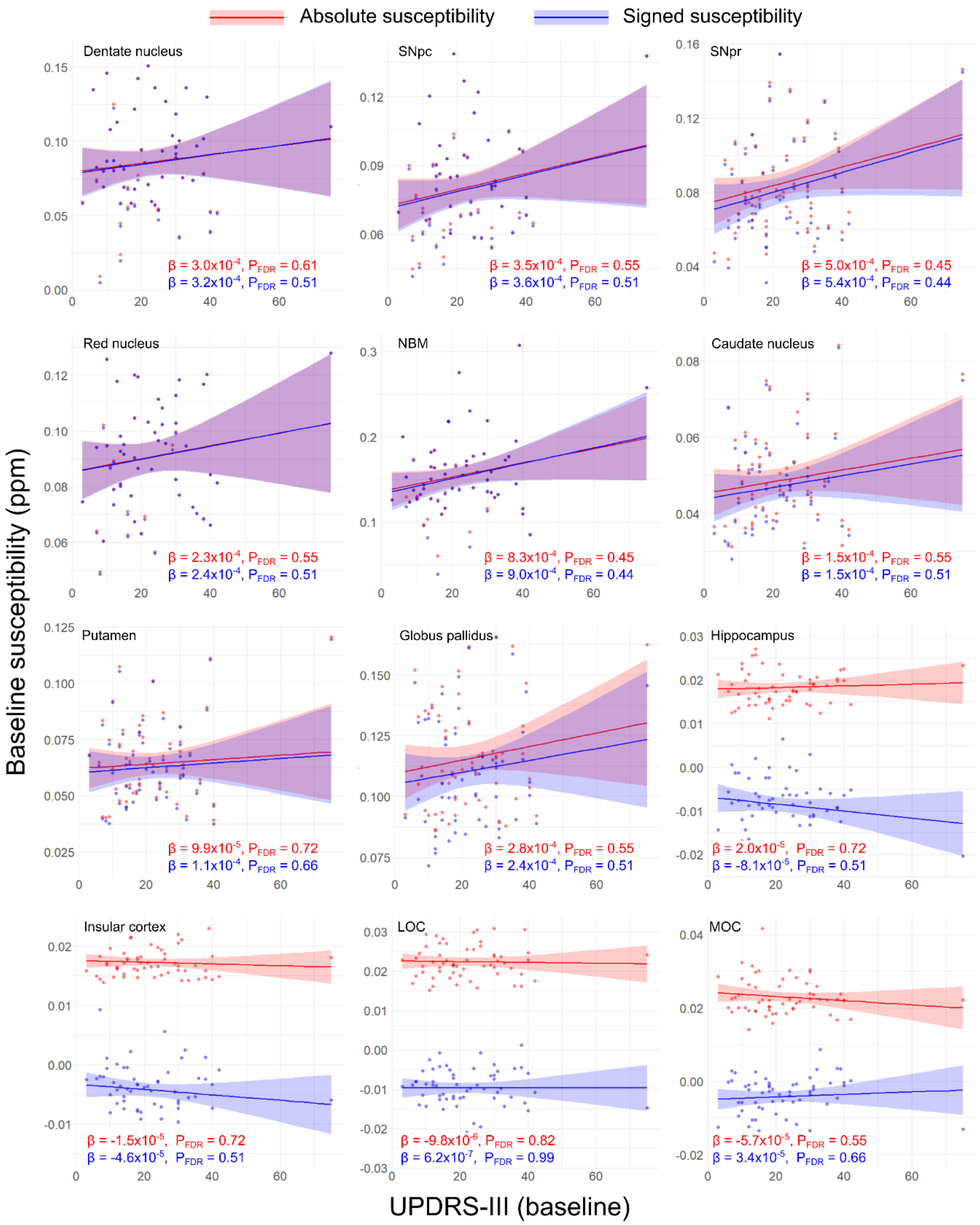

Supplementary Figure 6 – Regional relationships between baseline magnetic susceptibility and baseline motor severity score in Parkinson's disease. Data and statistics relating to ROI mean absolute susceptibility are shown in red, and those relating to ROI mean signed susceptibility are shown in blue. Results are adjusted for age at baseline and sex. FDR-corrected p-values (P_{FDR}) are presented. β is the linear model coefficient associated with MDS-UPDRS-III score. SNpc/pr = substantia nigra pars compacta / pars reticulata; NBM = nucleus basalis of Meynert; MOC = medial orbitofrontal cortex; LOC = lateral orbitofrontal cortex. MDS-UPDRS-III = Movement Disorders Society Unified Parkinson's Disease Rating Scale part III.

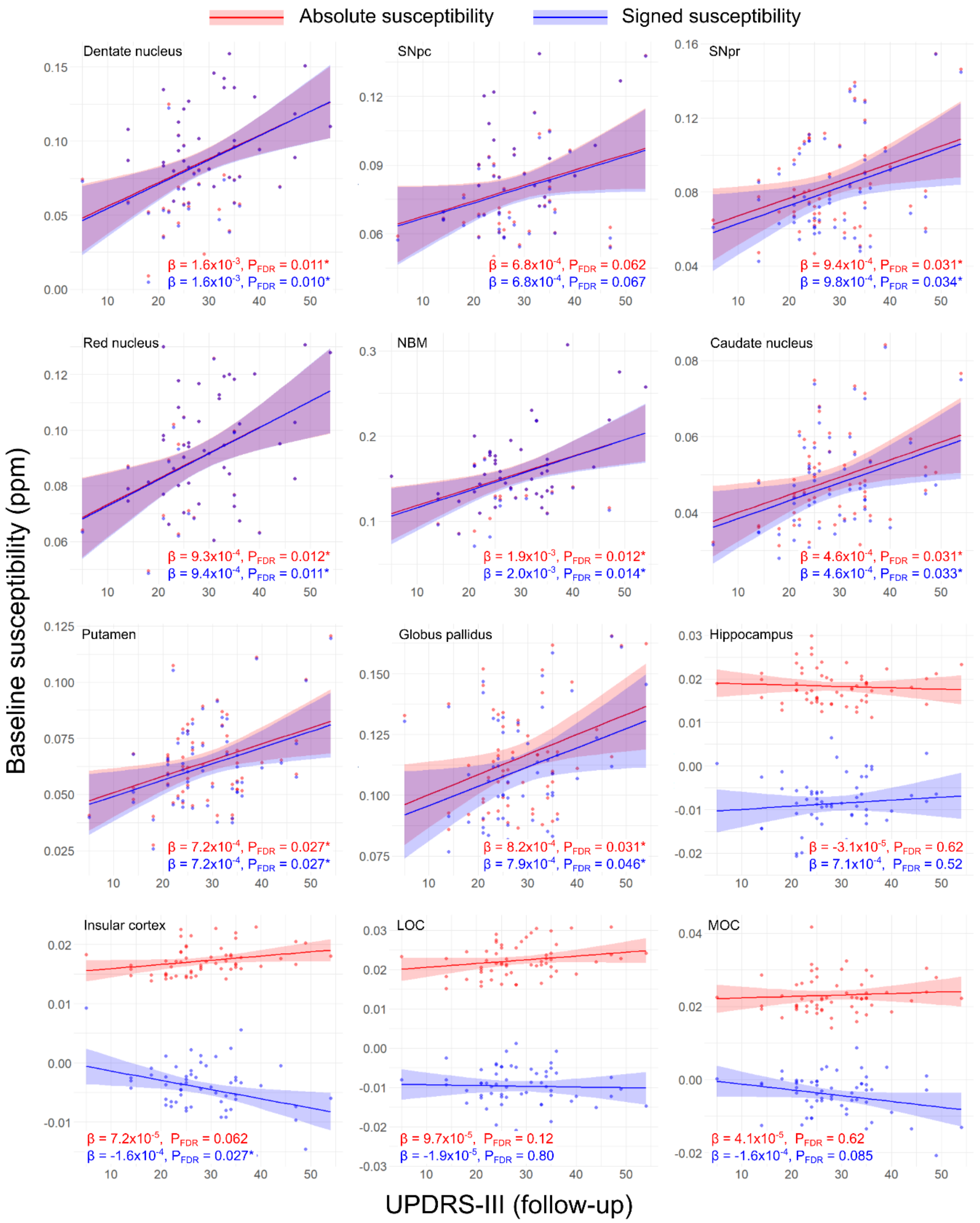

Supplementary Figure 7 – Regional relationships between follow-up magnetic susceptibility and follow-up motor severity score in Parkinson's disease. Data and statistics relating to ROI mean absolute susceptibility are shown in red, and those relating to ROI mean signed susceptibility are shown in blue. Results are adjusted for age at follow-up and sex. FDR-corrected p-values (P_{FDR}) are presented, with asterisks indicating significant interactions at $P_{FDR} < 0.05$. β is the linear model coefficient associated with MDS-UPDRS-III score. SNpc/pr = substantia nigra pars compacta / pars reticulata; NBM = nucleus basalis of Meynert; MOC = medial orbitofrontal cortex; LOC = lateral orbitofrontal cortex. MDS-UPDRS-III = Movement Disorders Society Unified Parkinson's Disease Rating Scale part III.

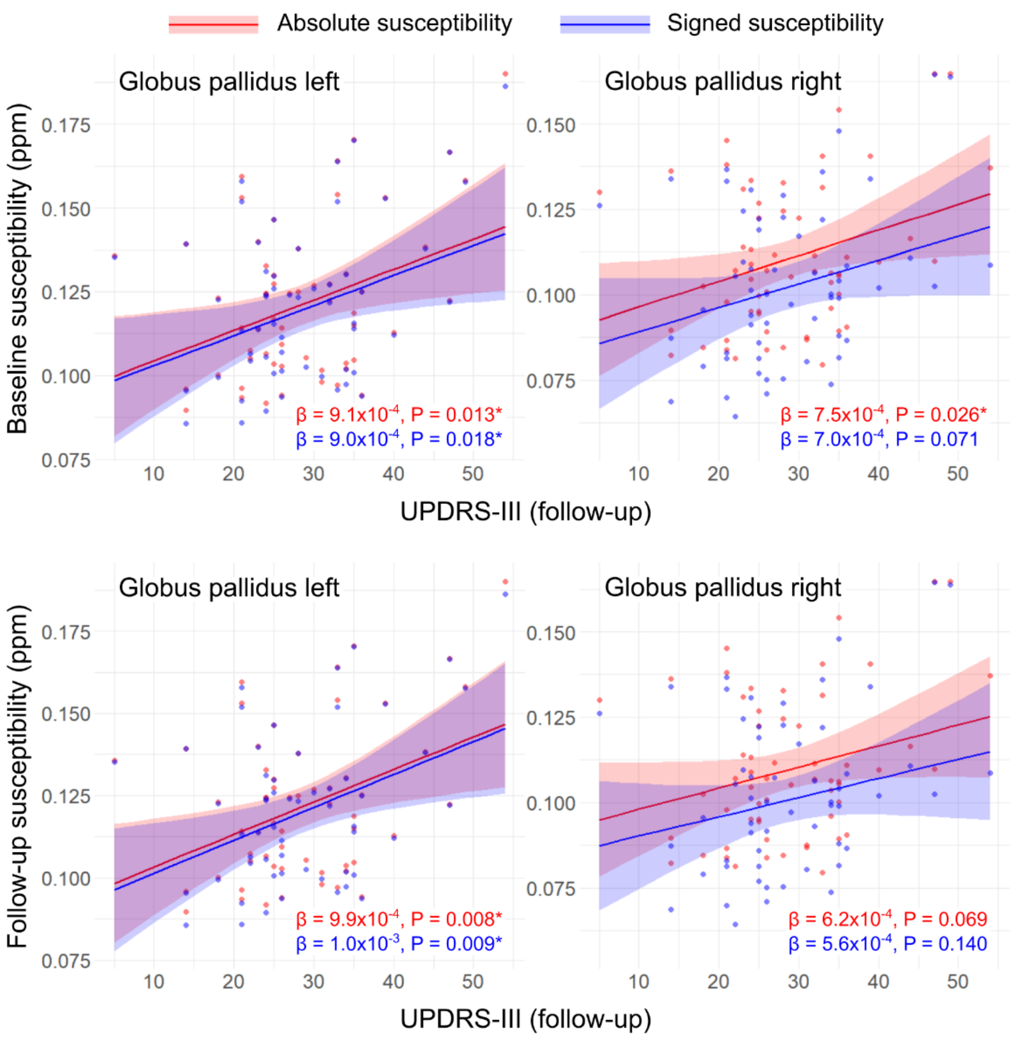

Supplementary Figure 8 – Asymmetry in the relationship between magnetic susceptibility in the globus pallidus and follow-up motor severity score in Parkinson's disease. Data and statistics relating to ROI mean absolute susceptibility are shown in red, and those relating to ROI mean signed susceptibility are shown in blue. Results are adjusted for age at follow-up and sex. Uncorrected p-values (P) are presented, with asterisks indicating significant interactions at $P < 0.05$ for illustrative purposes. β is the linear model coefficient associated with MDS-UPDRS-III score. Not that the relationship appears to be driven by the left globus pallidus. MDS-UPDRS-III = Movement Disorders Society Unified Parkinson's Disease Rating Scale part III.

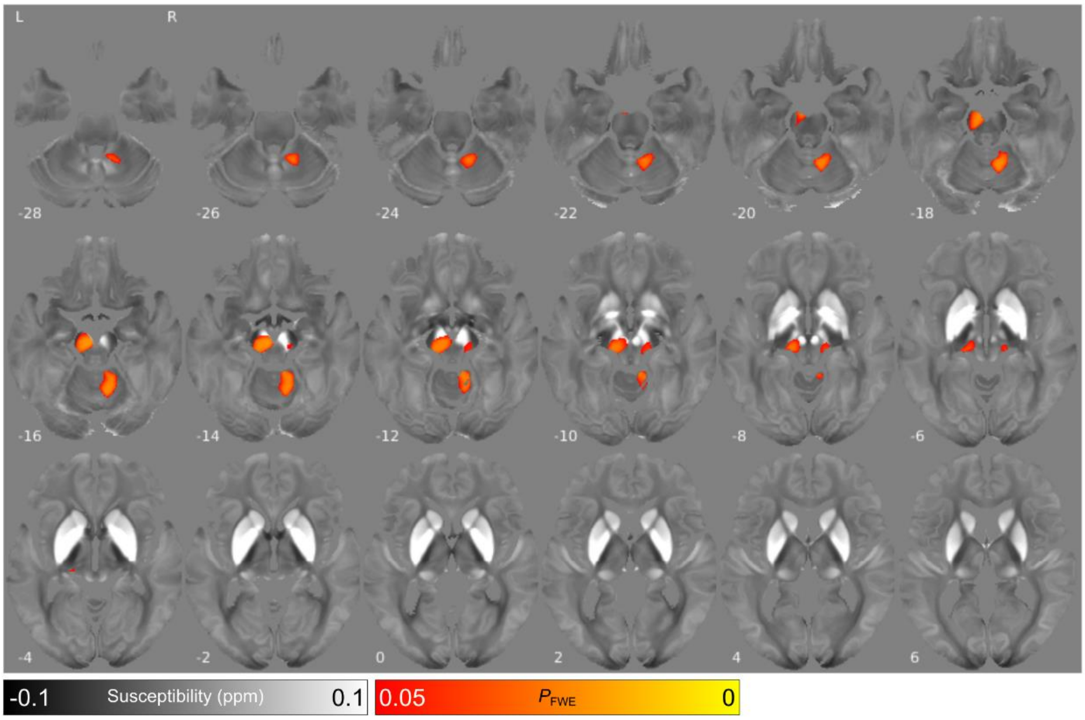

Supplementary Figure 9 – Relationship between absolute magnetic susceptibility and disease duration at follow-up in Parkinson's disease course, in a whole brain analysis. Whole brain analysis is adjusted for age at follow-up and sex. Red/yellow clusters represent voxels where a significant positive relationship was seen between absolute QSM and follow-up disease duration at FWE-corrected P<0.05. Note significant increases in susceptibility in the substantia nigra bilaterally and the left red nucleus. Results are overlaid on the study-wise QSM template in MNI152 space, and numbers represent axial slice location in MNI152 space.

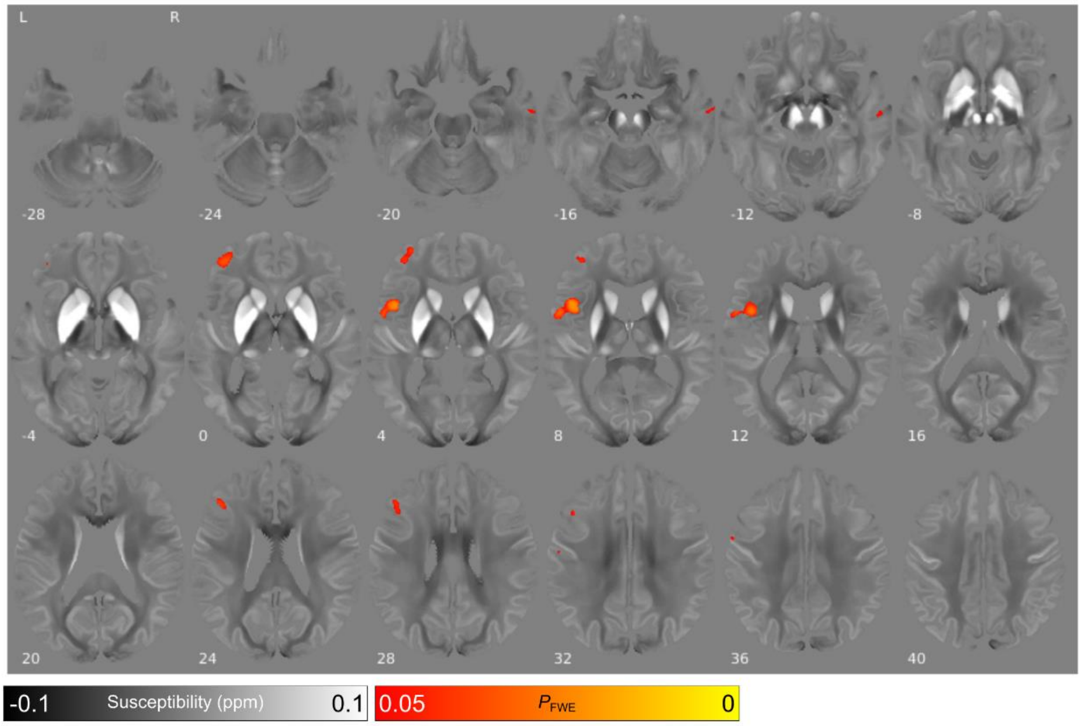

Supplementary Figure 10 – Changes in absolute magnetic susceptibility over time in Parkinson's disease at whole brain. Whole brain analysis is adjusted for age at baseline, sex and time between scans. Red/yellow clusters represent voxels where absolute QSM was significantly higher at follow-up at FWE-corrected P<0.05. Results are overlaid on the study-wise QSM template in MNI152 space, and numbers represent axial slice location in MNI152 space.

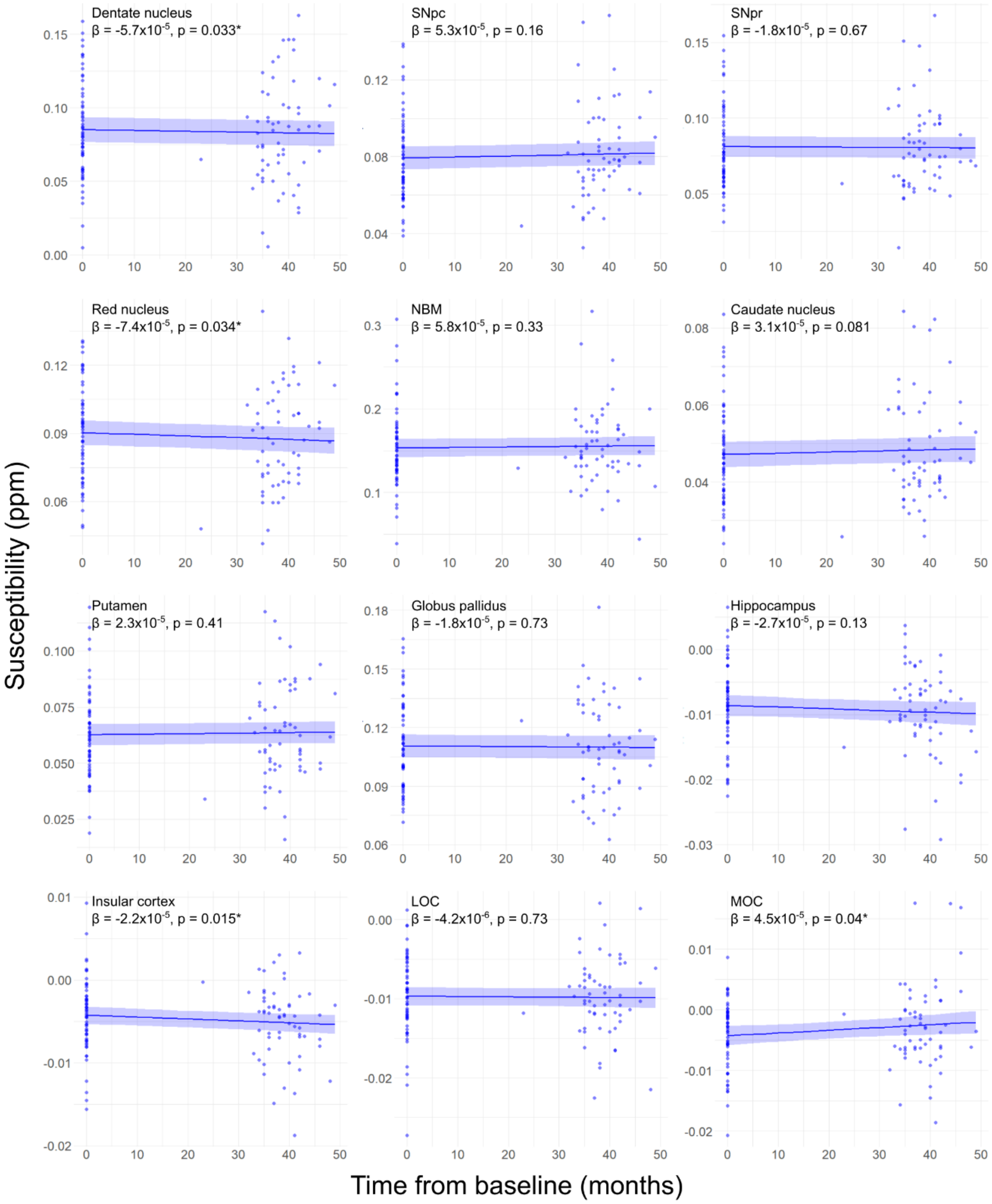

Supplementary Figure 11 - Results of linear mixed modelling showing regional change in magnetic susceptibility over time in Parkinson's disease. Change in susceptibility is modelled by fixed effects for time to follow-up (months), age at baseline and sex, and a random intercept effect per subject. β is the coefficient of the fixed effect for time to follow-up on susceptibility. Uncorrected P values indicate the significance of the effect of follow-up time on susceptibility, adjusted for age at baseline, sex and subject. Full ROI statistics can be seen in Supplementary Table 3. SNpc/pr = substantia nigra pars compacta / pars reticulata; NBM = nucleus basalis of Meynert; MOC = medial orbitofrontal cortex; LOC = lateral orbitofrontal cortex.

References

1. Thomas GEC, Leyland LA, Schrag AE, Lees AJ, Acosta-Cabronero J, Weil RS. Brain iron deposition is linked with cognitive severity in Parkinson's disease. Journal of Neurology, Neurosurgery and Psychiatry 2020;91(4):418-425.
2. Griswold MA, Jakob PM, Heidemann RM, et al. Generalized autocalibrating partially parallel acquisitions (GRAPPA). Magnetic Resonance in Medicine 2002;47(6):1202-1210.
3. Mori S, Wu D, Ceritoglu C, et al. MRICloud: Delivering high-throughput MRI neuroinformatics as cloud-based software as a service. Computing in Science and Engineering 2016;18(5):21-35.
4. Li X, Chen L, Kutten K, et al. Multi-atlas tool for automated segmentation of brain gray matter nuclei and quantification of their magnetic susceptibility. NeuroImage 2019;191:337-349.
5. Acosta-Cabronero J, Cardenas-Blanco A, Betts MJ, et al. The whole-brain pattern of magnetic susceptibility perturbations in Parkinson's disease. Brain 2017;140(1):118-131.
6. Yekutieli D, Benjamini Y. Resampling-based false discovery rate controlling multiple test procedures for correlated test statistics. Journal of Statistical Planning and Inference 1999;82(1-2):171-196.
